# Quantitative Susceptibility Mapping-Based Microscopy of Magnetic Resonance Venography (QSM-mMRV) for *In Vivo* Morphologically and Functionally Assessing Cerebromicrovasculature in Rat Stroke Model

**DOI:** 10.1371/journal.pone.0149602

**Published:** 2016-03-14

**Authors:** Meng-Chi Hsieh, Ching-Yi Tsai, Min-Chiao Liao, Jenq-Lin Yang, Chia-Hao Su, Jyh-Horng Chen

**Affiliations:** 1 Graduate Institute of Biomedical Electronics and Bioinformatics, National Taiwan University, Taipei 106, Taiwan; 2 Molecular Imaging Center, National Taiwan University, Taipei 106, Taiwan; 3 Department of Electrical Engineering, National Taiwan University, Taipei 106, Taiwan; 4 Institute for Translational Research in Biomedicine, Kaohsiung Chang Gung Memorial Hospital, Kaohsiung 833, Taiwan; 5 Department of Biomedical Imaging and Radiological Sciences, National Yang Ming University, Taipei 112, Taiwan; Henry Ford Health System, UNITED STATES

## Abstract

Abnormal cerebral oxygenation and vessel structure is a crucial feature of stroke. An imaging method with structural and functional information is necessary for diagnosis of stroke. This study applies QSM-mMRV (quantitative susceptibility mapping-based microscopic magnetic resonance venography) for noninvasively detecting small cerebral venous vessels in rat stroke model. First, susceptibility mapping is optimized and calculated from magnetic resonance (MR) phase images of a rat brain. Subsequently, QSM-mMRV is used to simultaneously provide information on microvascular architecture and venous oxygen saturation (SvO_2_), both of which can be used to evaluate the physiological and functional characteristics of microvascular changes for longitudinally monitoring and therapeutically evaluating a disease model. Morphologically, the quantification of vessel sizes using QSM-mMRV was 30% smaller than that of susceptibility-weighted imaging (SWI), which eliminated the overestimation of conventional SWI. Functionally, QSM-mMRV estimated an average SvO_2_ ranging from 73% to 85% for healthy rats. Finally, we also applied QSM to monitor the revascularization of post-stroke vessels from 3 to 10 days after reperfusion. QSM estimations of SvO_2_ were comparable to those calculated using the pulse oximeter standard metric. We conclude that QSM-mMRV is useful for longitudinally monitoring blood oxygen and might become clinically useful for assessing cerebrovascular diseases.

## Introduction

Stroke is the leading cause of long-term disability, also one of the commonest causes of mortality in aging countries [1]. Abnormal structure and blood oxygen saturation (SO_2_) of cerebral microvessels (diameter: ≤ 100 μm) [2] is a critical feature of stroke. Characterizing unusual microvascular change and extraordinary SO_2_ might be useful for the diagnosis and the prognosis of stroke [1,3]. Thus, measuring cerebral blood oxygen saturation might be necessary for an accurate diagnosis, to predict disease outcomes, and to monitor the treatment response in stroke.

The most commonly used noninvasive methodologies of medical imaging in clinical and experimental neuroscience for assessing the cerebral microvessels in cerebrovascular diseases like stroke, glioma, and vascular malformation are computed tomography angiography (CTA) and magnetic resonance angiography (MRA). Although CTA with a contrast agent can rapidly and accurately detect the structure of blood vessels [4], it has the potential negative side affect of ionizing radiation. In contrast, MRA-based techniques, such as time-of-flight (TOF)-MRA and contrast-enhanced (CE)-MRA, are not radioactive. TOF-MRA is sensitive to the fast-flowing signals in arteries and depends on the motion of water protons [5]. However, TOF-MRA is limited to measuring small cerebral vessels (venules, arterioles, and capillaries) because of slow-flowing signals in the cerebral microvessels. CE-MRA uses gadolinium (Gd)-based contrast agents to detect these slow-flowing signals [6]. Nonetheless, CE-MRA might not satisfy the long acquisition time required for high-resolution MRA application because it has a short intravascular half-life and rapidly redistributes into the extracellular space.

Deoxyhemoglobin, however, provides natural contrast enhancement. Based on this advantage, susceptibility-weighted imaging (SWI) has been proposed for visualizing venous vascular architecture and has provided structural information for more than a decade [7]. Furthermore, SWI combines MR magnitude and phase images, and it is more sensitive for detecting magnetic substances such as deoxyhemoglobin, hemorrhage, iron, etc. Moreover, SWI is also widely used clinically to visualize and diagnose venous vascular malformations, stroke, and traumatic brain injuries. It has also been used to longitudinally assess ischemic vessel size in a rat stroke model [8]. Although it can characterize vascular structure, SWI cannot provide functional information about blood vessels.

To quantify vascular information, previous studies assessed venous oxygen saturation (SvO_2_) with the relaxation time T_2_* [9,10]. However, T_2_* is not a high-specificity index because it depends on the measurement conditions of B_0_ inhomogeneity, on the relaxation time T_2_ (without the effect of B_0_ inhomogeneity), and on the properties of blood vessels. Additionally, T_2_* produces inconsistent results under various B_0_s because of the dependence between T_2_ and B_0_. Conversely, the intrinsic susceptibility of hemoglobin is a potential index for measuring SvO_2_. Based on susceptibility measurements, others have shown that MR phase images can be used to estimate SvO_2_ in the brain tissue of humans [11–15] and rodents [16,17]. Nevertheless, using MR phase imaging to quantify SvO_2_ is vessel-orientation dependent, and MR phase images show apparent blooming artifacts.

Recently, a novel approach called quantitative susceptibility mapping (QSM), based on MR phase imaging, is being used to quantify MR images with magnetic susceptibility without blooming artifacts [18–24]. In addition, QSM has be used to assess the SvO_2_ in healthy human brains [20,25–28] and to detect decreases in the SvO_2_ in patients with cerebral ischemic stroke [29]. Although the QSM technique has been used quantify to SvO_2_ in humans, it has not been used to quantify cerebral SvO_2_ in rat stroke model. Characterizing the cerebral SvO_2_ with QSM in rat stroke model facilitates the understanding of mechanism in brain disorder. Thus, applying QSM to rat stroke model is crucial for neuroscience research.

The present study aimed to investigate the feasibility of using QSM for assessing cerebral SvO_2_ on stroke in rat. A 3D high-resolution gradient-echo (GRE) image with a spatial resolution of 100 × 100 × 100 μm^3^ was used to generate the QSM. QSM processing was optimized from single-echo GRE images for rat brain venography, which included phase unwrapping, background field removal, and dipole inversion. The influence of choosing different values of QSM parameter was investigated. The cerebral microvessels of a healthy rat were visualized and the SvO_2_ quantified after QSM reconstruction. To demonstrate the abilities of QSM, we also compared it with the conventional SWI method. Finally, QSM was used for longitudinal monitoring of post-stroke revascularization on days 3–10 after reperfusion.

## Materials and Methods

### Susceptibility Calculation Based on Phase Information

The effects of magnetic susceptibility can be observed in the image phase information obtained using the gradient echo sequence. Denote the obtained image phase map as φ, with k-space representation φ(k). Similarly, let χ and χ(k) respectively represent the spatial distribution and Fourier domain representation of the susceptibility map. The relationship between the measured phase and underlying susceptibility map are expressed as follows [30]:

$$\varphi (k)=-\chi (k)\cdot D(k)\cdot TE\cdot \gamma \cdot B_{0}$$ (1)

where D(k), TE, γ, and B_0_ are the dipole kernel in the Fourier domain, echo time, gyromagnetic ratio of ^1^H, and main magnetic field, respectively. The dipole kernel is denoted as $D(k)=1/3-k_{z}^{2}/\left(k_{x}^{2}+k_{y}^{2}+k_{z}^{2}\right)$, where k_x_, k_y_, and k_z_ respectively represent the vectors of k-space in the x-, y-, and z-axes. The center of D(k) was set to zero. Theoretically, the susceptibility map can be obtained from the phase map simply by inverting the dipole kernel D(k):

$$\chi (k)=-\varphi (k)/\left[D(k)\cdot TE\cdot \gamma \cdot B_{0}\right]$$ (2)

However, D(k) vanishes in the conical surface region along the magic angle (54.7°) defined by $2k_{z}^{2}=k_{x}^{2}+k_{y}^{2}$ [31]. Thus, χ(k) at that conical surface region cannot be determined. In addition, the non-uniform distribution of D(k) results in noise propagation after this inversion. Both factors contribute to the well-known streaking artifacts commonly observed in QSM [19,31].

### Regularized Approach for QSM

To obtain a stable solution to this ill-posed problem, several nonlinear L1 regularized methods have been reported for artifact-free QSM [21,24,32–35]. In this study, we used an L1 regularization with magnitude prior, which is similar to the popular method called morphology enabled dipole inversion (MEDI) [22,24], which improves conventional L1 regularization to eliminate both the underestimation of the susceptibility value and the streaking artifacts. The L1 regularization with magnitude prior involves the following minimization:

$$\begin{array}{cccc} \chi^{*}=\frac{1}{2}{\left\|b-F^{-1}DF\chi \right\|}_{2}^{2}+\lambda \cdot {\left\|WG\chi \right\|}_{1} & with & W=\left[\begin{array}{c} W_{x}\\ W_{y}\\ W_{z} \end{array}\right], & G=\left[\begin{array}{c} G_{x}\\ G_{y}\\ G_{z} \end{array}\right] \end{array}$$ (3)

where χ* is the regularized susceptibility value, b is the internal field perturbation, F is the 3D fast Fourier transform operator, λ is the Lagrange parameter, W is the prior information of a binary low-gradient mask (edges were set to zeros, and all others were ones) in three dimensions acquired by simple thresholding of the magnitude gradient, and G is the gradient operator in three dimensions. To solve the minimization problem, the steepest gradient descent method was applied [36]. For comparison, the conventional L1 method was used by minimizing $\chi^{*}=\frac{1}{2}{\left\|b-F^{-1}DF\chi \right\|}_{2}^{2}+\lambda \cdot {\left\|G\chi \right\|}_{1}$ with no weighting factor (W).

### SvO_2_ Calculation with Susceptibility

Measuring susceptibility difference from QSM makes it possible to quantify SvO_2_ values of interest based on the relationship between susceptibility difference and SvO_2_:

$$\Delta \chi_{vein-tissue}=\Delta \chi_{do}\cdot Hct\cdot (1-SvO_{2})$$ (4)

where Δχ_vein-tissue_ is the estimated susceptibility difference between vein and gray matter, Δχ_do_ = 0.18 ppm (cgs) is the susceptibility difference between fully deoxygenated and fully oxygenated blood [37], and Hct is the hematocrit coefficient, which is 0.4 in the venous vessels of rat brains [38]. Arteries were assumed to be fully oxygenated with an SO_2_ of 100% in this study [39].

### Ethics Statement

All animal experimental procedures in this study were approved by the Institutional Animal Care and Use Committee of National Taiwan University and Kaohsiung Chang Gung Memorial Hospital, and were in compliance with the guidelines for animal care and use set forth by that Committee. These criteria have been established by the Institutional Animal Care and Use Committee, which recognizes that euthanasia is sometimes necessary prior to the scheduled end of a study, either because of unanticipated complications, or because of the protocol itself. Furthermore, the euthanasia is necessary when animal meet one of the criterion, including (1) weight loss, (2) inappetence, (3) weakness or inability to obtain feed or water, (4) moribund state, (5) infection, and (6) signs of severe organ system dysfunction and non-responsive to treatment, or with a poor prognosis as determined by a veterinarian. The rat was euthanized by 100% CO_2_ at the end of experiment or one of the criterion that described above-mentioned.

### Middle Cerebral Artery Occlusion (MCAO)

To study post-stroke rehabilitation, QSM was used in a rat model of middle cerebral artery occlusion (MCAO) stroke. The detailed procedures of middle cerebral artery (MCA) reperfusion are described elsewhere [40]. Male Sprague-Dawley rats 7–9 weeks old were intraperitoneally injected with sodium pentobarbital anesthetic (50 mg/kg-bw [body weight]). The right eye-to-ear area was then shaved, and the rats were placed in a prone position on a warming pad at 37°C and incubated with positive-pressure ventilation (0.2 mL/sec) with oxygen using a small animal ventilator (SAR-830/A; CWE, Ardmore, PA, USA). A 1.5-cm incision was made on the scalps of the anesthetized rats, at the midpoint between the right eye and right ear. The temporalis muscle was separated to expose the zygoma and squamosal bones. A dental drill was used to make a 2-mm^2^ burr hole 1 mm rostral to the anterior junction of the zygoma and the squamosal bones. The dura mater was carefully pierced with a microsurgical needle. The exposed MCA was carefully isolated and ligated for 60 min using 10.0 surgical sutures (Johnson & Johnson Medical, Somerville, NJ, USA) to induce ischemic stroke in the cortex of the right hemisphere. Isoflurane (2%) was on hand in case the rats woke up during surgery. When the MCA ligation was complete, the common carotid arteries (CCAs) on both sides were ligated using aneurysm clips. The ligations on both the CCAs and the MCA were loosened after 60 min. All procedures were completed in two h. All of animals were treated carprofen as analgesic with the dosage of 5 mg/Kg (s.c., BID) after the post-operative of MCAO. The brain was dissected and incubated with triphenyl tetrazolium chloride (TTC) to determine the ischemic infarct area. For immunohistochemistry, the brains were obtained and then equilibrated in 20% sucrose at 4°C after they had been reperfused with 4% paraformaldehyde in PBS.

### Calculating the Ischemic Infarct Area Using TTC Staining

We used TTC staining to determine the infarct area in the brain tissue sections of the Stroke group (MCA-ligated) rats. Three or 10 days after the MCA had been reperfused, the rats were euthanized with isoflurane (100% CO_2_) and their brains were removed. The brains were dissected and collected, frozen at −20°C for 5 min, cut into 2-mm coronal sections, and then stained with 2% TTC (T8877; Sigma-Aldrich, St. Louis, MO, USA) in PBS for 8 min at 37°C. The stained sections were transferred to 4% paraformaldehyde for immersion fixation for 24 h, dehydrated in 30% sucrose, and then photographed.

### Measuring the Pulse Oxygen Saturation (SpO_2_) in the Infarcted and Non-Infarcted Areas of the Brain Tissue Sections of the Stroke Rat

The SpO_2_ was measured with a pulse oximeter system (Radical; Masimo Corp., Irvine, CA, USA). After the rats had been anesthetized with 1.5–2% isoflurane gas mixture (20% O_2_; 80% N_2_), they were placed on the animal holder, and the sensor was placed above the infarct and normal areas of the rat brain, respectively. The SpO_2_ was recorded at 30-s intervals for 5 min. The SpO_2_ measurement used here was to examine the trend of the oxygenation level over time in the Stroke group.

### MR Data Acquisition for Control Rats

The Sprague-Dawley rats (n = 6; male; 8–10 weeks old; weight: 303 ± 4.2 g) were anesthetized with 2% isoflurane flowing in a gas mixture (O_2_, 20%; N_2_, 80%). Their respiration rate was monitored and maintained at 50 breaths per min (bpm) with a monitoring and gating system (SA Instruments, Stony Brook, NY, USA). Rectal temperatures (36 ± 0.5°C) were maintained using a warm-water circulation system. The MR experiments were done using a 7-T animal MRI scanner (BioSpin 70/30; Bruker GmbH, Ettlingen, Germany). A 7-cm linear birdcage volume coil was used for signal excitation, and a 4-channel phased array was used for signal reception. T_2_*-WI was acquired using a 3D-GRE first-order flow compensation sequence that prevents signal dephasing of the laminar flow of blood in vessels. The imaging parameters were FOV = 38.4 × 25.6 × 12.8 mm^3^, MTX = 384 × 256 × 128, voxel size = 100 × 100 × 100 μm^3^, TR/TE = 65.5/15 ms, bandwidth = 25 kHz, and scan time = 36 min. To obtain an acceptable phase contrast between veins and surrounding tissue, the TE was set as the T_2_* value of the deoxygenated vessel [41]. The flip angle was set at about 15°-20° to obtain the optimal phase contrast between the grey and white matter [39]. The large-scale B_0_ inhomogeneity was minimized by region of interest (ROI)-based shimming (provided with the system).

### MR Data Acquisition for the MCAO Rat Stroke Model

A 9.4-T animal MRI scanner (BioSpin 94/20; Bruker) was used to visualize the brain tissue sections of the Sprague-Dawley rats in the Stroke (n = 6) and the Control groups. A 7-cm quadrature birdcage volume coil was used for signal excitation, and a 4-channel phased array was used for signal reception. T_2_*-WI was acquired using a 3D-GRE sequence with first-order flow compensation. The imaging parameters were FOV = 38.4 × 25.6 × 12.8 mm^3^, MTX = 384 × 256 × 128, voxel size = 100 × 100 × 100 μm^3^, TR/TE = 50/12 ms, bandwidth = 23 kHz, and scan time = 28 min.

### MR Data Processing

Multichannel MR raw data were reconstructed using MATLAB (The MathWorks, Natick, MA, USA), and then separated into magnitude and phase images. The magnitude images of the individual channels of the coil array were combined using the sum-of-squares method [42] (Figure AA in S1 File), and the phase images were assembled using complex summation [43] (Figure AB in S1 File). Subsequently, the combined magnitude and phase images were used for QSM and SWI reconstruction.

**Fig 1:**
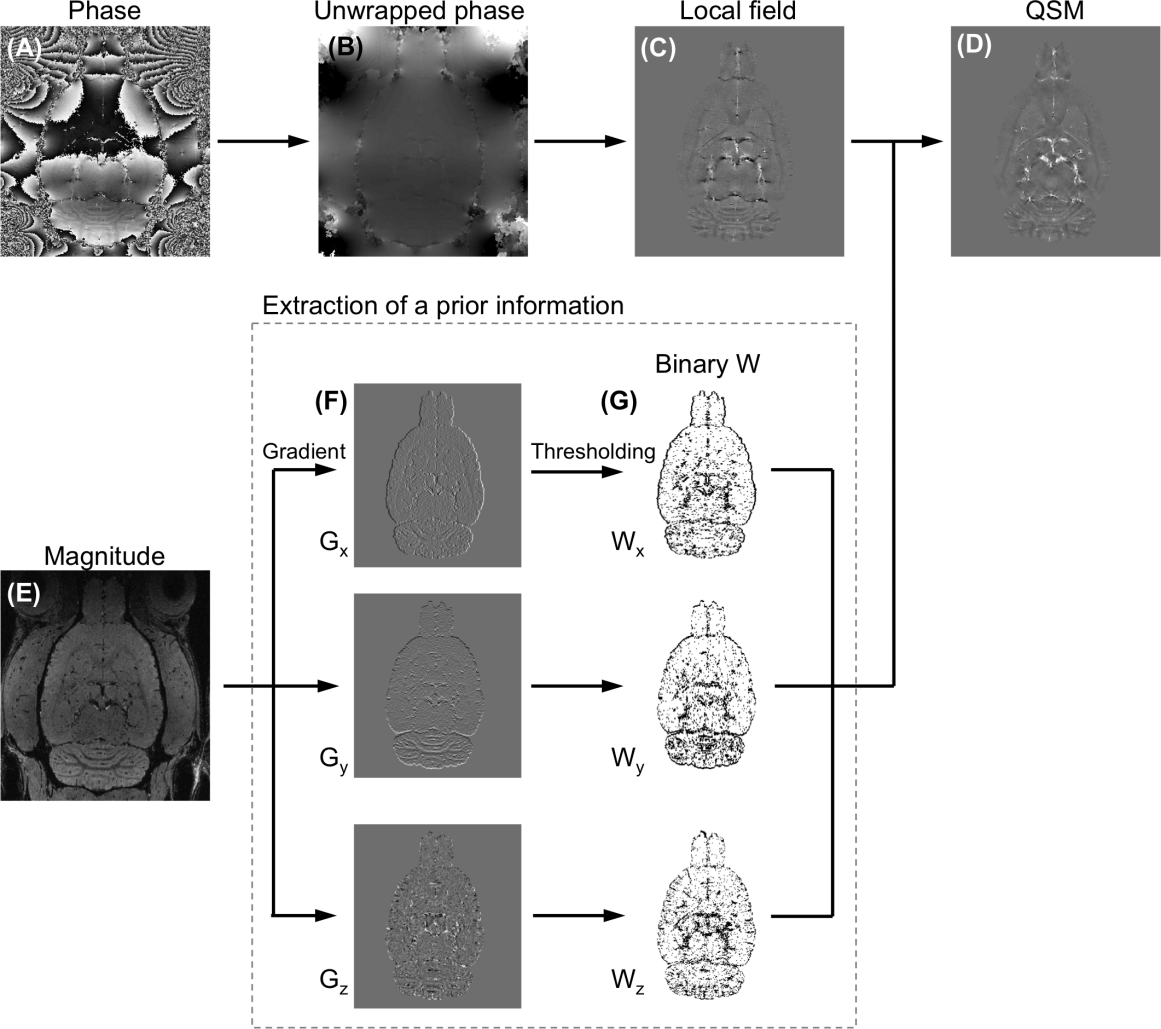
The steps used in the QSM process. (A) Phase image, (B) unwrapped phase image, (C) internal field map, and (D) QSM in rat brain region. (E-G) The steps used in the extraction of prior information from magnitude images.

**Fig 2:**
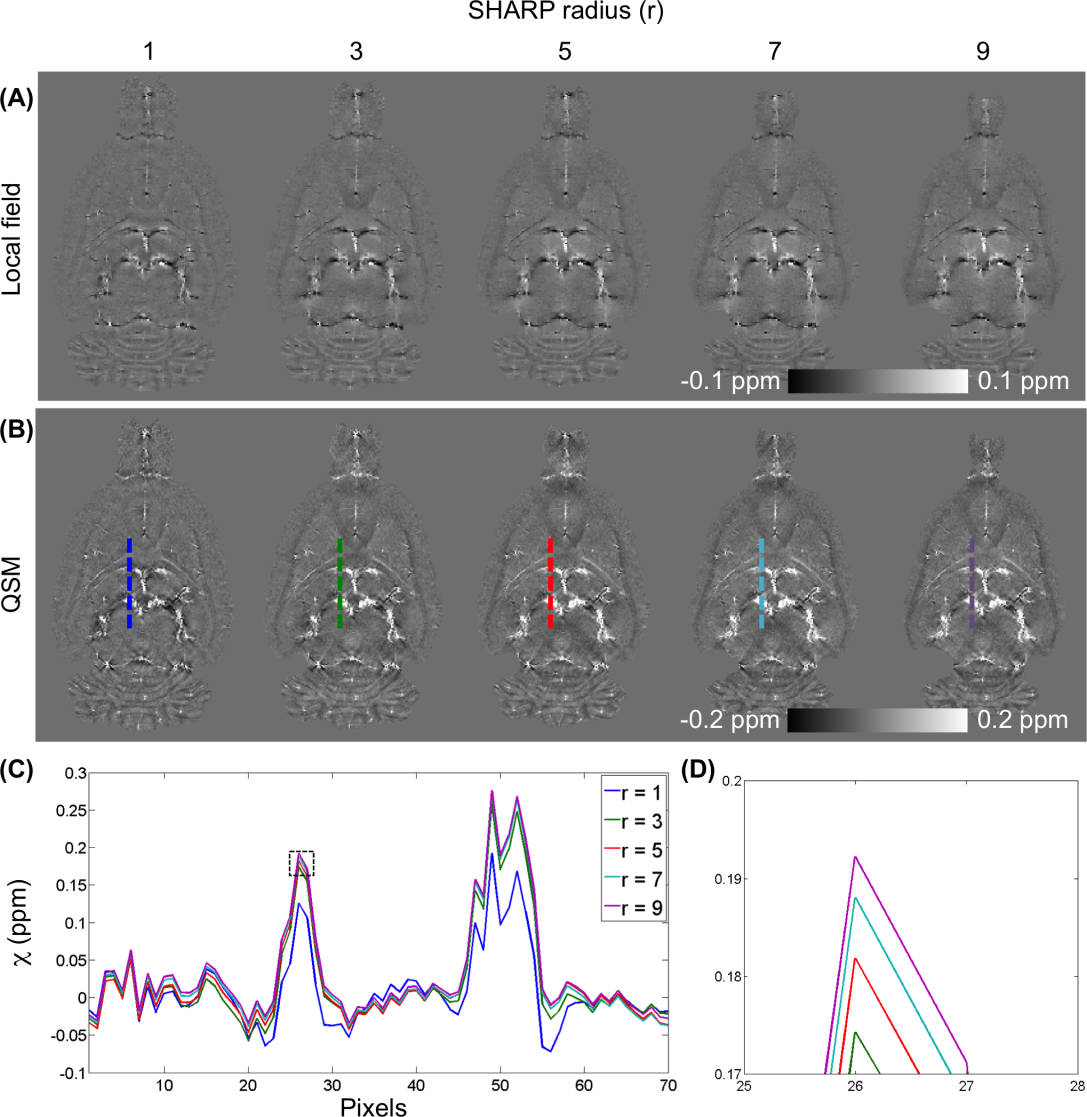
Selection of the radius of the sophisticated harmonic artifact reduction for phase data (SHARP) filtering. (A) Local fields and (B) QSMs (L1 regularization with λ = 10^−3^) calculated by varying the radius of SHARP filtering from 1 to 9 in 2-voxel steps. (C) Difference in line profile of reconstructed QSM among various radii. In this figure, path-based phase unwrapping is used. (D) The enlargement of the rectangular (dotted line) in (C).

Fig 1 shows an outline of the procedures of QSM reconstruction. First, phase aliasing is resolved using a phase-unwrapping algorithm. Phase wrapping appears commonly in high-field imaging when large off-resonance is present and when TE is relatively long. Two phase-unwrapping methods, path-based in a spatial domain [44,45] and Laplacian-based in a Fourier domain [32], have been proposed to resolve phase wrap around 2π. Although the Laplacian-based method is fast, it results significant errors in the vicinity of the vessels. Hence, the best 3D path-based method was used for QSM-mMRV [45]. Second, the unwrapped phase φ (Fig 1B) was normalized to field perturbation ΔB = Δφ/(γ∙TE∙B_0_). The magnitude images of the rat brains were manually segmented using MRIcro [46] to generate a brain mask applied to ΔB. Third, a background field induced by air-tissue susceptibility differences and imperfect shimming was then removed to obtain the internal field b (Fig 1C) using sophisticated harmonic artifact reduction for phase data (SHARP) [47]. The advantage of the SHARP method over conventional high-pass filtering is that it can preserve the low-frequency component of the local phase. The optimal local field using SHARP filtering was accomplished using truncated singular value decomposition with a radius of 3 voxels (300 μm), a shell thickness of 1 voxel (100 μm), and a truncation value of 0.05 as described elsewhere [47]. The radius was determined by examining the line profile from the reconstructed QSM (L1 regularization with λ = 10^−3^) and varying the value from 1 to 9 voxels in steps of 2 (Fig 2). A radius of 3 voxels was used in this study. Because SHARP filtering cannot be computed for voxels that are less than a radius away from the ROI border, the local field is available only on a modified ROI that is smaller than the original ROI. In addition, using a radius of 1 voxel caused over-filtering. Moreover, the truncation value was determined empirically by varying the value from 0.025 to 0.15 in steps of 0.025 and visually inspecting the resultant local field distribution. Finally, the QSM (Fig 1D) was calculated from b by minimizing the magnitude prior L1 method as Eq (3) with steepest gradient decent algorithm of 10 iterations. The Lagrange multiplier λ determines the smoothness term (‖WGχ‖_1_ or ‖Gχ‖_1_) and data consistency term ($\frac{1}{2}{\left\|b-F^{-1}DF\chi \right\|}_{2}^{2}$) of the reconstructed susceptibility map such that larger values of λ yield smoother image results than do smaller ones. In this study, the λ = 10^−1.2^ was selected for the optimal QSM according to the L-curve criterion [48] by varying the λ logarithmically between 10^−4^ and 10^0.6^ (Figure B in S1 File). The binary weighting mask was derived from the magnitude gradient in three directions (threshold was set to 0.03). For comparison, the conventional L1 regularization QSM was also optimized using the L-curve criterion (Figure C in S1 File).

**Fig 3:**
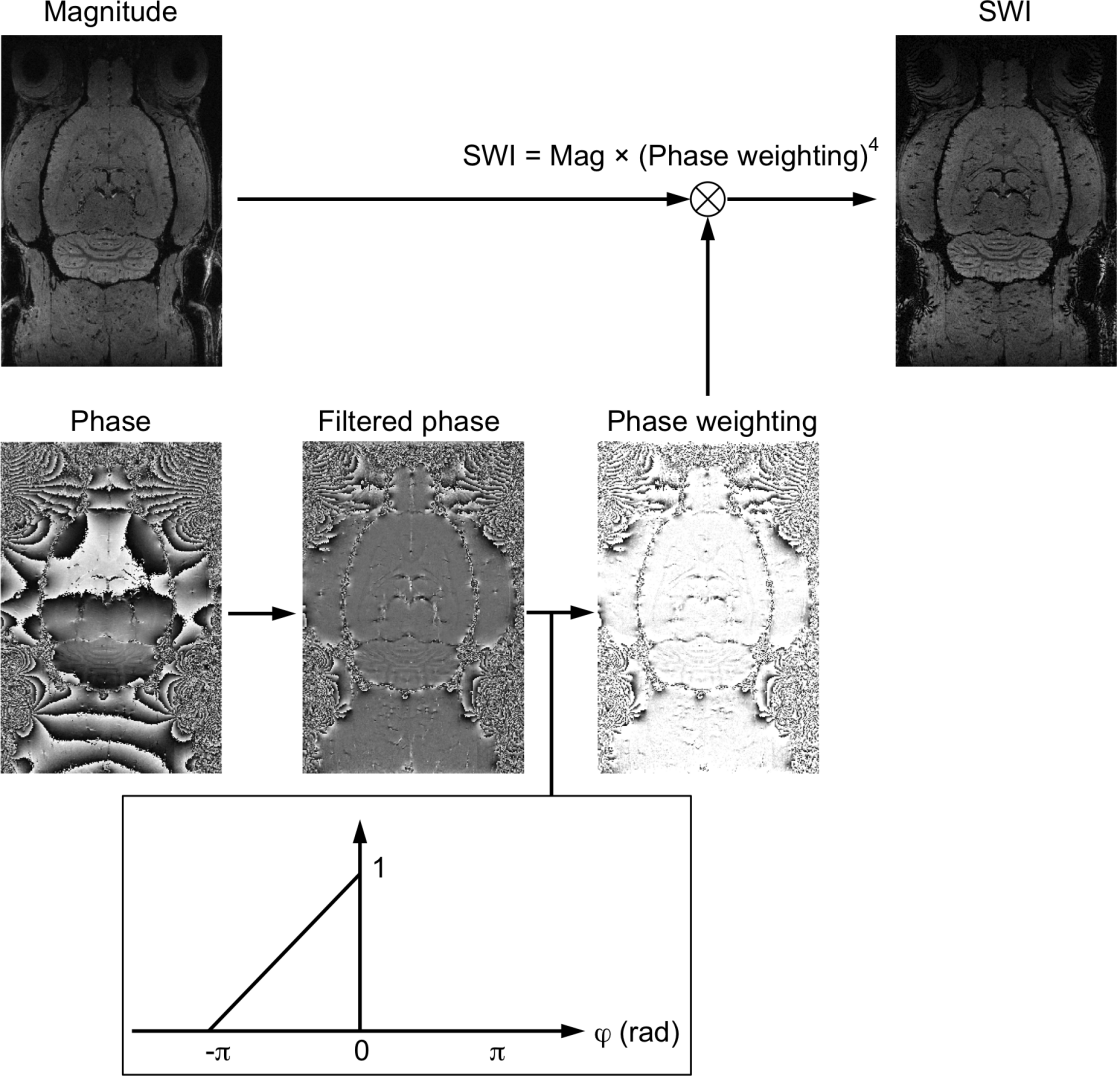
Flowcharts of processing steps of susceptibility-weighted images (SWI). SWI combines both magnitude and a filtered phase map with a multiplicative relationship to enhance image contrast.

For a comparison of vein detection, SWI was reconstructed using the same 3D-GRE data with a Hann (Hanning) window size of 64 [7] (Fig 3).

### Image Registration and Statistical Analysis of MRI Data

To quantitatively compare the susceptibility differences of cerebral veins between rats, the susceptibility value of the cortex was selected as a reference [49]. Subsequently, the brain regions were registered to a Sprague-Dawley rat brain atlas [50] using the linear registration algorithm in FSL FLIRT software with affine transformations [51,52]. First, the veins were extracted (threshold was set to χ > 0.05 ppm). Subsequently, the susceptibility values were measured using an average intensity projection (AIP) of a 25-slice QSM (2.5-mm effective coverage). Seven ROIs of veins, including the intracortical penetrating venule, middle internal frontal vein (MIF), longitudinal hippocampal vein (LHIV), medial collicular vein (MCOLV), thalamostriate vein (THSV), the great cerebral vein (GCV) of Galen, and straight sinus (STS) were manually drawn and extracted. All measured data are mean ± standard deviation (SD). A coronal slice of cortical brain was selected to compare the differences in measured vessel sizes between SWI and QSM. In studying post-stroke revascularization, a 2.5-mm-thick SWI minimum intensity projection (mIP) and 2.5-mm-thick QSM MIP were used to compare. Significance was set at *p* < 0.05 (two-tailed *t* test).

## Results

### Path-Based and Laplacian-Based Unwrapping Algorithms Compared

**Fig 4:**
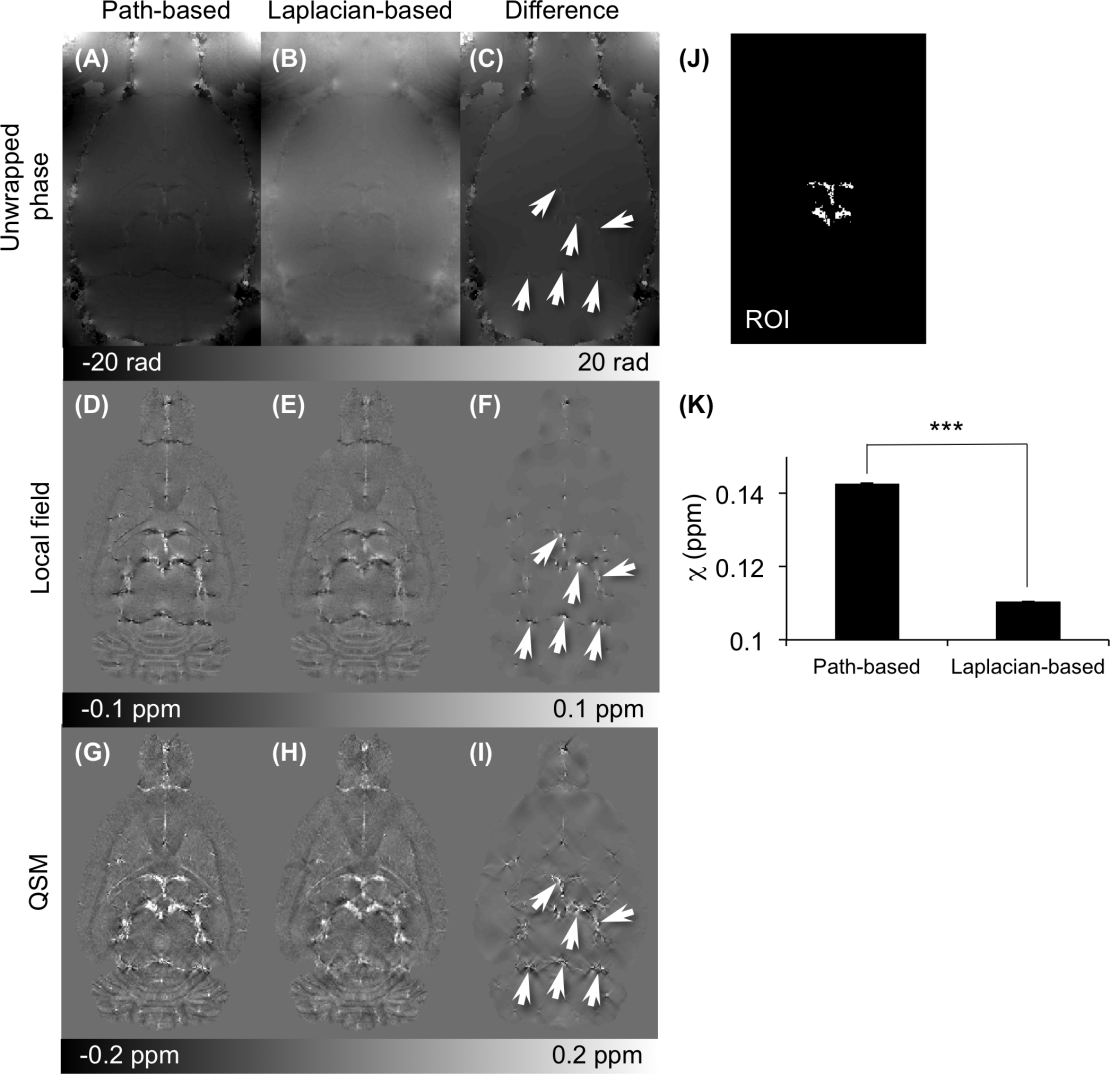
Comparison of quantitative susceptibility map (QSM) based on path-based and Laplacian-based phase unwrapping. Images computed using 3D path-based and Laplacian-based phase unwrapping are presented in the first and second columns from the left. The third column depicts the difference in the images in the first two columns. (A-C) Comparison of the unwrapped phase images with path-based and Laplacian-based algorithms. (D-F) Comparison of the local field (SHARP filtering with radius of 3 voxels). (G-I) Comparison of reconstructed QSM (L1 regularization with λ = 10^−3^). The arrows point to regions where significant differences between the images in the left and middle columns were observed. (K) Comparison of the measured susceptibility values in vessels from (J) the region-of-interest (ROI) (***p < 0.001).

Fig 4 compares the 3D path-based unwrapping and the Laplacian-based unwrapping algorithm as well as the local field images and susceptibility maps (under-regularized L1 QSM with λ = 10^−3^) from the unwrapped phase images. The “Difference” images of the Unwrapped phase (Fig 4C), Local field (Fig 4F), and susceptibility (Fig 4I) maps appear visible difference close to the THSV, LHIV, and TRS vessels. The Laplacian-based unwrapping method yielded an underestimation of the susceptibility in vessels (*p* < 0.001) over the path-based unwrapping (Fig 4K). In addition, the accuracy of the local field acquired by the two unwrapping methods was confirmed and compared using numerical simulations (Figure C in S2 File).

### Influence of the Selection of the Lagrange Parameter λ

**Fig 5:**
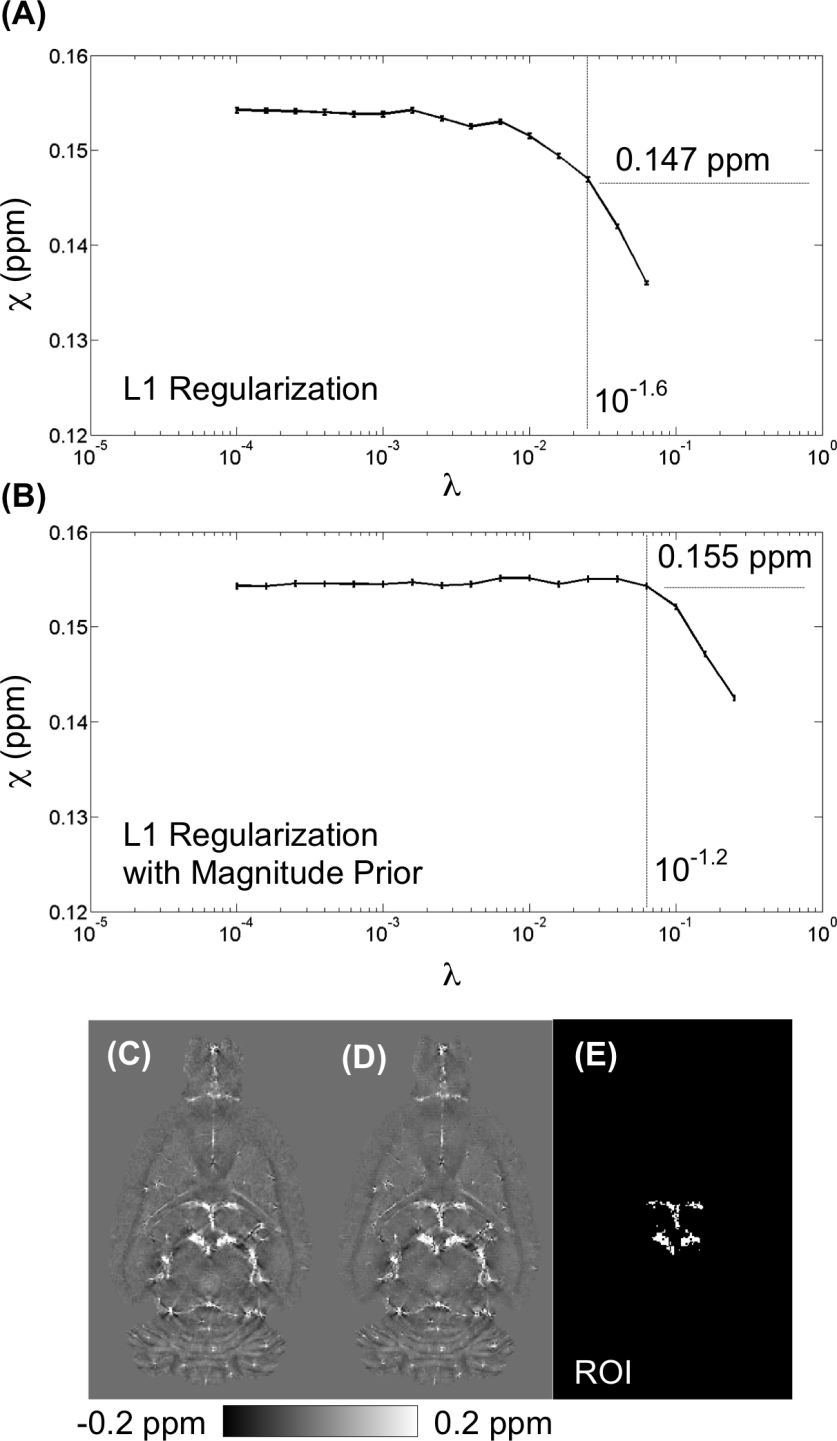
Influence of the Selection of the Lagrange parameter λ. Plot of the measured susceptibility value from region-of-interest (E) across various λ by (A) L1 regularization and (B) L1 regularization with magnitude prior. At optimal weighting of λ = 10^−1.6^, L1 regularization resulted in 0.147 ppm. In contrast, at optimal weighting of λ = 10^−1.2^, L1 regularization with magnitude prior resulted in 0.155 ppm.

The magnitude prior L1 and conventional L1 regularizations were both optimized using the L-curve criterion (Figures B and C in S1 File), and the Lagrange parameters were selected as 10^−1.2^ and 10^−1.6^ for the magnitude prior L1 and the L1 regularized QSM, respectively. Fig 5 illustrates the effect of the Lagrange parameter on the susceptibility value from the reconstructed QSM in the vein. Fig 5A and 5B compare the optimal L1 QSM (λ = 10^−1.6^), which yielded an underestimation in susceptibility of 0.147 ppm relative to 0.155 ppm using the optimal magnitude prior L1 algorithm (λ = 10^−1.2^).

### Using QSM to Quantitatively Visualize Veins

**Fig 6:**
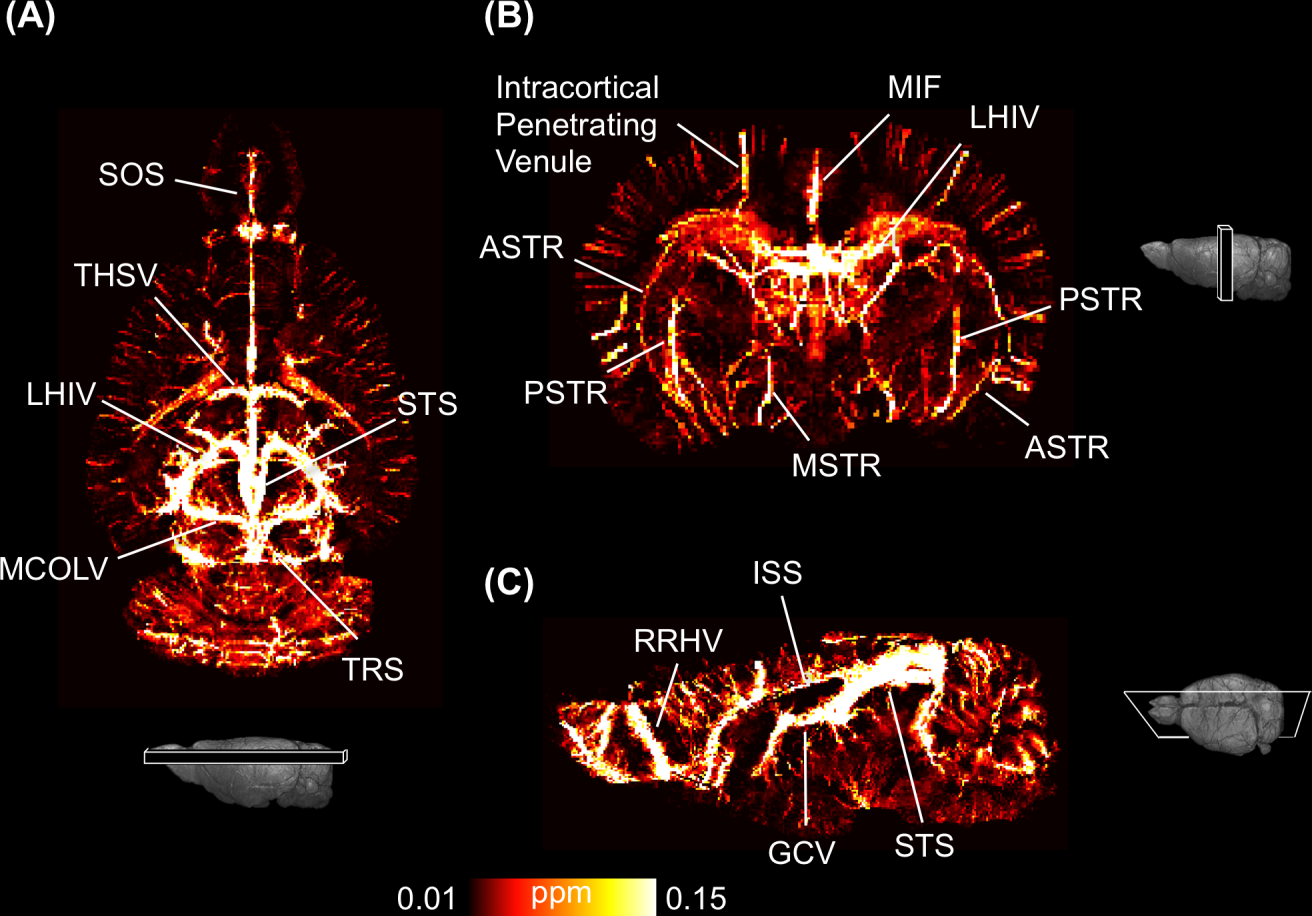
Quantitative visualization of QSM of a normal rat brain in three orthogonal views. Veins in the cortical and internal brain are indicated. (A) A 2.5-mm-thick maximum intensity projection (MIP) in coronal view, (B) axial view, and (C) sagittal view. The major veins are labeled, including the great cerebral vein (GCV) of Galen, intracortical penetrating venule, inferior sagittal sinus (ISS), longitudinal hippocampal vein (LHIV), medial collicular vein (MCOLV), superior olfactory sinus (SOS), superior sagittal sinus (SSS), straight sinus (STS), thalamostriate vein (THSV), transverse sinus (TRS), middle internal frontal vein (MIF), anterior striate vein (ASTR), posterior striate vein (PSTR), medial striate vein (MSTR), and rostral rhinal vein (RRHV).

The 3D high-resolution QSM was reconstructed from a T_2_*-WI using the optimized regularized approach. Fig 6 shows the maximum intensity projection (MIP) of QSM calculated from a 2.5-mm-thick brain slice and reveals various veins and venules in each of the three orthogonal planes. These veins were validated and identified using the cerebral vascular atlas [53]: the great cerebral vein (GCV) of Galen, intracortical penetrating venule, inferior sagittal sinus (ISS), longitudinal hippocampal vein (LHIV), medial collicular vein (MCOLV), superior olfactory sinus (SOS), superior sagittal sinus (SSS), straight sinus (STS), thalamostriate vein (THSV), transverse sinus (TRS), middle internal frontal vein (MIF), anterior striate vein (ASTR), posterior striate vein (PSTR), medial striate vein (MSTR), and rostral rhinal vein (RRHV). The average susceptibilities of seven major veins (intracortical penetrating venule, MIF, LHIV, MCOLV, THSV, GCV, and STS) were measured from AIP of QSM, and their SvO_2_ levels were calculated (Table 1). The *in vivo* SvO_2_ levels in veins ranged from 82.38 ± 3.51% to 90.82 ± 0.75%. QSM allowed (1) microvessels to be visualized at a resolution of 100 × 100 × 100 μm^3^ and (2) SvO_2_ to be quantified.

**Table 1:** Estimated SvO_2_ of seven regions of interest from seven Control group rats (%).

B_0_	7-T						9.4-T	
Vein	Rat 1	Rat 2	Rat 3	Rat 4	Rat 5	Rat 6	Rat 7	Mean Across Subjects
Intracortical venule	90.53	91.01	92.50	90.95	90.04	90.35	90.38	90.82 ± 0.75
MIF	88.82	90.26	90.79	90.58	90.93	88.61	89.02	89.86 ± 0.93
LHIV	83.78	85.48	88.14	84.75	83.36	83.45	83.86	84.69 ± 1.57
MCOLV	84.66	88.25	88.55	85.47	85.38	88.38	88.53	87.03 ± 1.63
THSV	85.62	88.01	90.09	86.25	86.35	87.76	87.28	87.34 ± 1.38
GCV	84.12	85.64	88.02	84.35	85.59	86.77	88.18	86.10 ± 1.51
STS	82.92	85.62	87.06	82.95	76.54	78.16	83.41	82.38 ± 3.51

MIF, middle internal frontal vein; LHIV, longitudinal hippocampal vein; MCOLV, medial collicular vein; THSV, thalamostriate vein; GCV, the great cerebral vein of Galen; STS, straight sinus.

### QSM and SWI Compared

**Fig 7:**
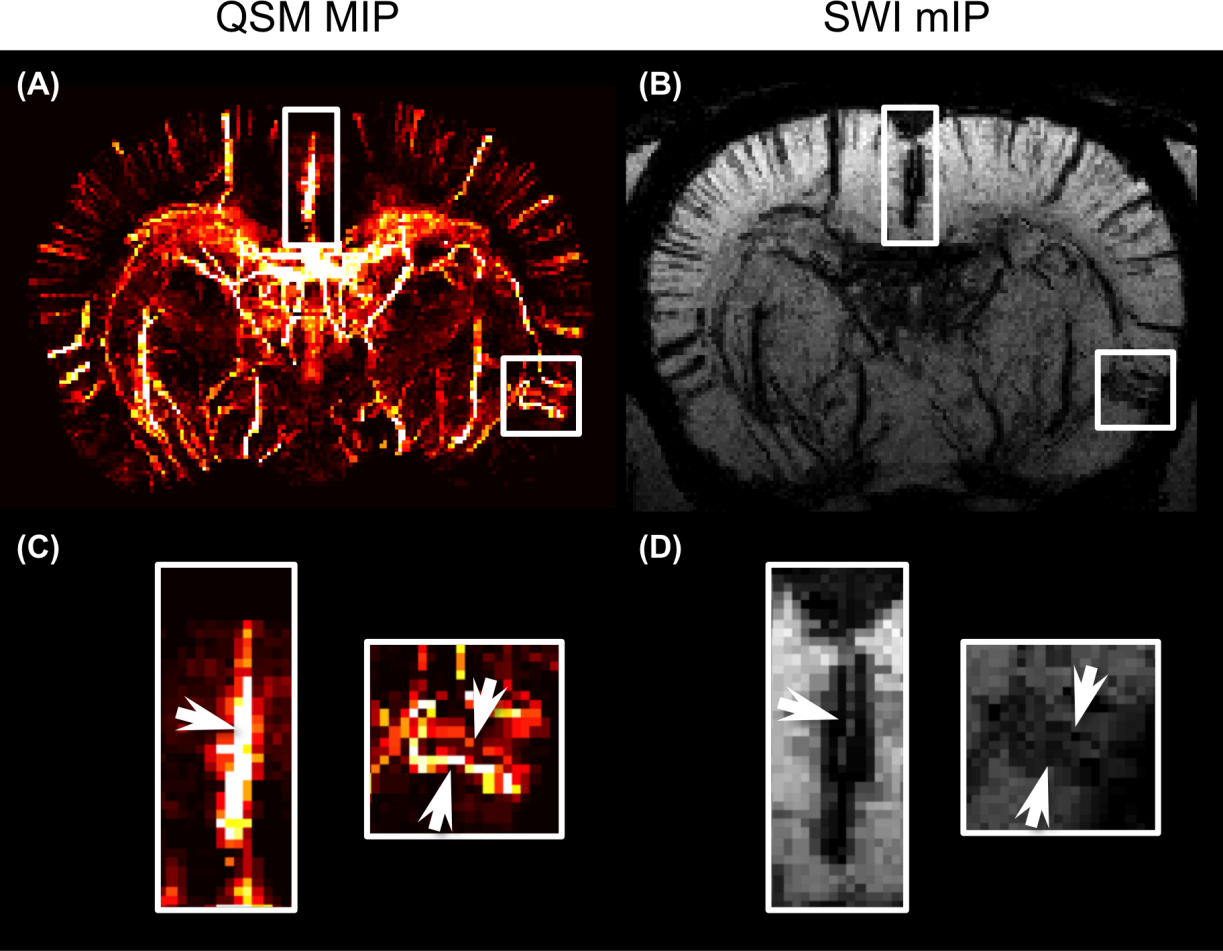
Comparison of QSM and susceptibility-weighted imaging (SWI). (A) A 2.5-mm-thick MIP of QSM in axial view. The cortex in dorsal and lateral brain are marked by the rectangles and magnified in (C). (B) A 2.5-mm-thick minimum intensity projection (mIP) of SWI with identical ROIs. (D) In the mIP of SWI, the vein has blooming artifacts and is difficult to identify. The arrows point to the significant difference between QSM and SWI.

The images obtained using QSM and SWI were compared using the same 3D-GRE data. Fig 7A and 7B show the respective QSM and SWI results for the same axial 2.5-mm-thick slice. The microvessels of the dorsal and lateral cortical areas were identified using both angiographic techniques because both used the same signal source: deoxyhemoglobin. However, SWI lacks quantitative information about the microvessels, but QSM provides the SvO_2_ of the cerebral vessels. Moreover, the QSM technique eliminates both the blooming artifacts and overestimation of vessel size, while QSM deconvolved the dipole kernel from the phase image (Fig 7C and 7D).

**Fig 8:**
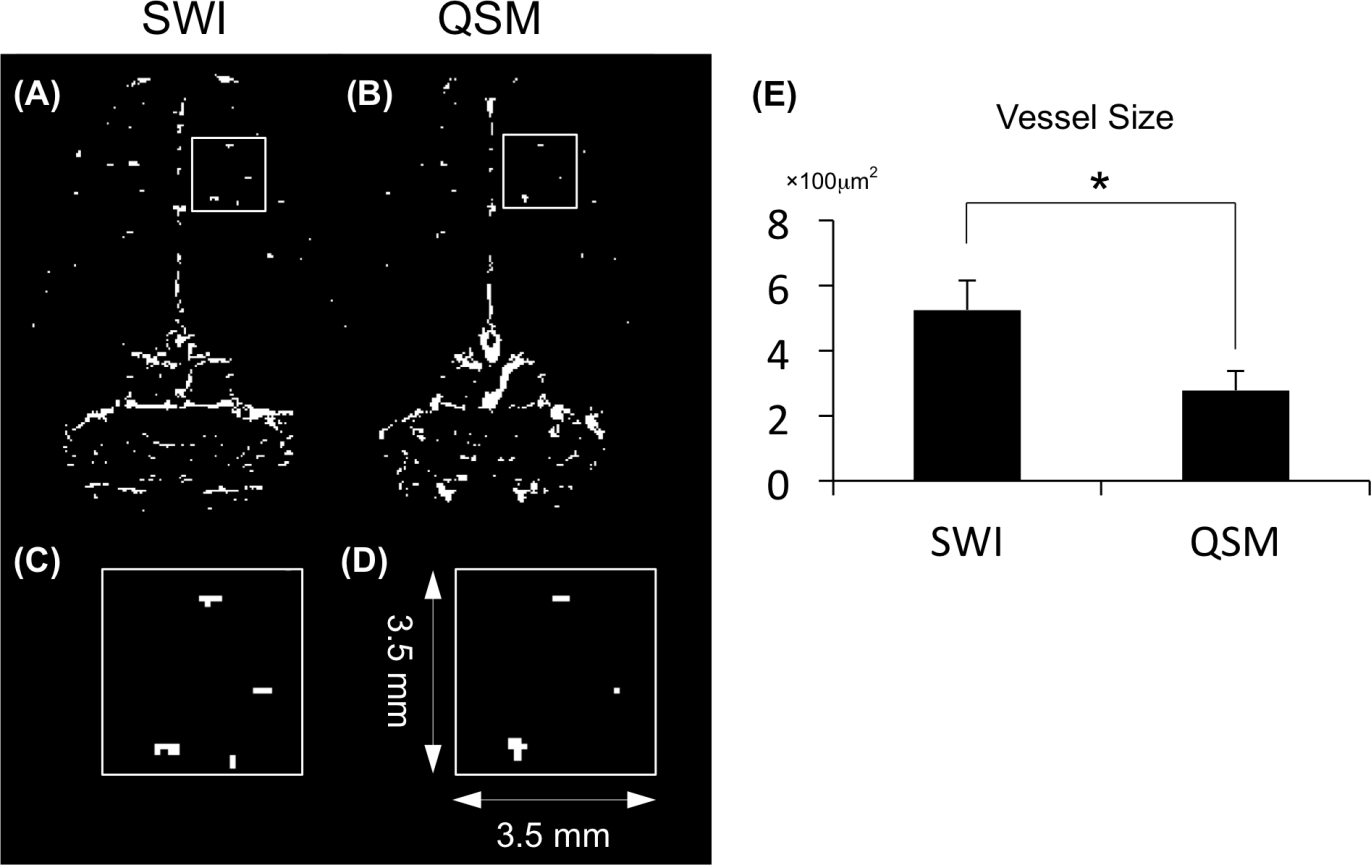
Illustration of differences in the intracortical penetrating vessels between SWI and QSM. (A) A coronal slice from SWI. (B) A coronal slice from QSM. (C) Magnified view of a 3.5 × 3.5-mm^2^ region of SWI marked by red rectangle in A. The bright signal represents the through-plane cortical vessels. (D) Fewer and smaller bright signals in QSM. (E) Quantification of vessel size using the two methods. (*p < 0.05).

SWI and QSM images of a coronal slice in the cortical region-show that the distribution of the intracortical vessels revealed by the two methods are consistent, no significant difference (*p* > 0.05) in vessel density between SWI (0.0036 ± 0.0011 pixel/mm^2^) and QSM (0.0031 ± 0.0008 pixel/mm^2^), and that the vessels appeared smaller in the QSM image (Fig 8A and 8B). Fig 8C and 8D show enlargements of the white-lined boxes in Fig 8A, 8B and 8E shows quantified vessel cross sectional areas. Quantitative analysis of intracortical venules shows that SWI estimated that the vessels were significantly (*p* < 0.05)—1.4 times—larger than estimated by QSM.

### Using QSM to Study Post-Stroke Rehabilitation

**Fig 9:**
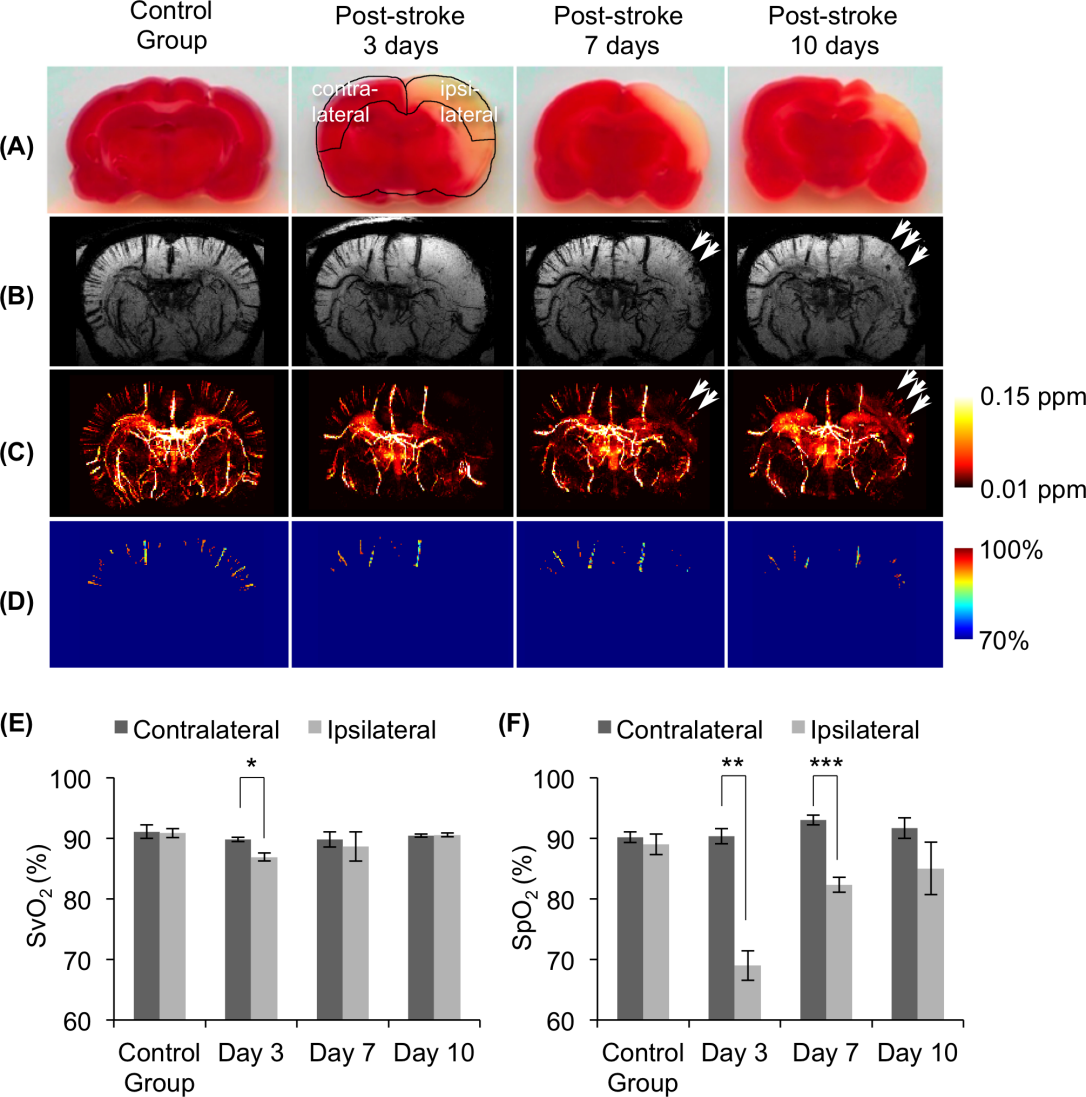
Detection of the rehabilitation of intracortical venules in the rat brain after stroke. (A) Triphenyl tetrazolium chloride (TTC)-stained slice for infarcted cortical area confirmation over time. (B) Representative of 2.5-mm-thick minimum intensity projection (mIP) of SWI over time. (C) Representative of 2.5-mm-thick average intensity projection (AIP) of QSM over time. (D) Representative of 2.5-mm-thick SvO_2_ map over time. The second figure in the first row illustrates the ROI selections on the contralateral (left) and ipsilateral (right) cortices. The arrows indicate angiogenesis at 7 and 10 days post-reperfusion. (E) (F) Comparison of SvO_2_ estimates and SpO_2_ measures on a post-stroke rat. (E) SvO_2_ estimates by QSM-mMRV and (F) SpO_2_ measures using the pulse oximeter on contralateral and ipsilateral cortices. (Mean and SD cross subjects are presented; *p < 0.05; **p < 0.01; ***p < 0.001).

QSM and SWI were used to study post-stroke rehabilitation in a Stroke group rat 3, 7, and 10 days after the MCAO had been reperfused. TTC staining shows that the infarcted cortical area shrank from day 3 to day 10 (Fig 9A). A 2.5-mm-thick axial view of mIP using SWI (Fig 9B) and QSM-estimated SvO_2_ maps (Fig 9D) clearly show variations in the cortical venules. The SvO_2_ estimations of the ipsilateral and contralateral vessels from day 3 to day 10 are shown in Fig 9E. The SvO_2_ level of the vessel on the ipsilateral-cortex was significantly lower than that of the vessel on the contralateral-cortex on day 3 (*p* < 0.05). After the reperfusion, however, the SvO_2_ level of the ipsilateral-cortex climbed, which indicated that the oxyhemoglobin had reached a plateau on day 7 and 10. This result was comparable with the SpO_2_ levels calculated using the pulse oximeter (Fig 9F).

## Discussion

In the present study, we applied QSM to assess cerebral SvO_2_ in rat stroke model. The magnitude prior L1-regularized QSM method was also optimized to provide an accurate estimation of susceptibility values and to suppress streaking artifacts. Relative to SWI, QSM-mMRV eliminates the blooming artifacts from the phase image, which reduces overestimations of vessel size and makes it easy to distinguish intracortical vessels. When used to longitudinally monitor rehabilitation, the proposed method showed the SvO_2_ changes in microvessels 3, 7, and 10 days post-stroke, which was comparable with the SpO_2_ measures using the pulse oximeter standard metric.

The path-based unwrapping algorithm was more reliable for QSM-mMRV than the Laplacian-based method. We found that the measured susceptibility in veins of the reconstructed QSM from the unwrapped phase using path-based unwrapping was significantly higher than the Laplacian-based unwrapping (Fig 4K). The numerical simulation also verified that the path-based unwrapping algorithm presented the true unwrapped phase in the spatial domain (Figure C in S2 File). Although studies [32,54–57] have reported using Laplacian-based unwrapping to successfully reconstruct QSM, it might cause an incorrect calculation near the vessels [32,54,55].

**Fig 10:**
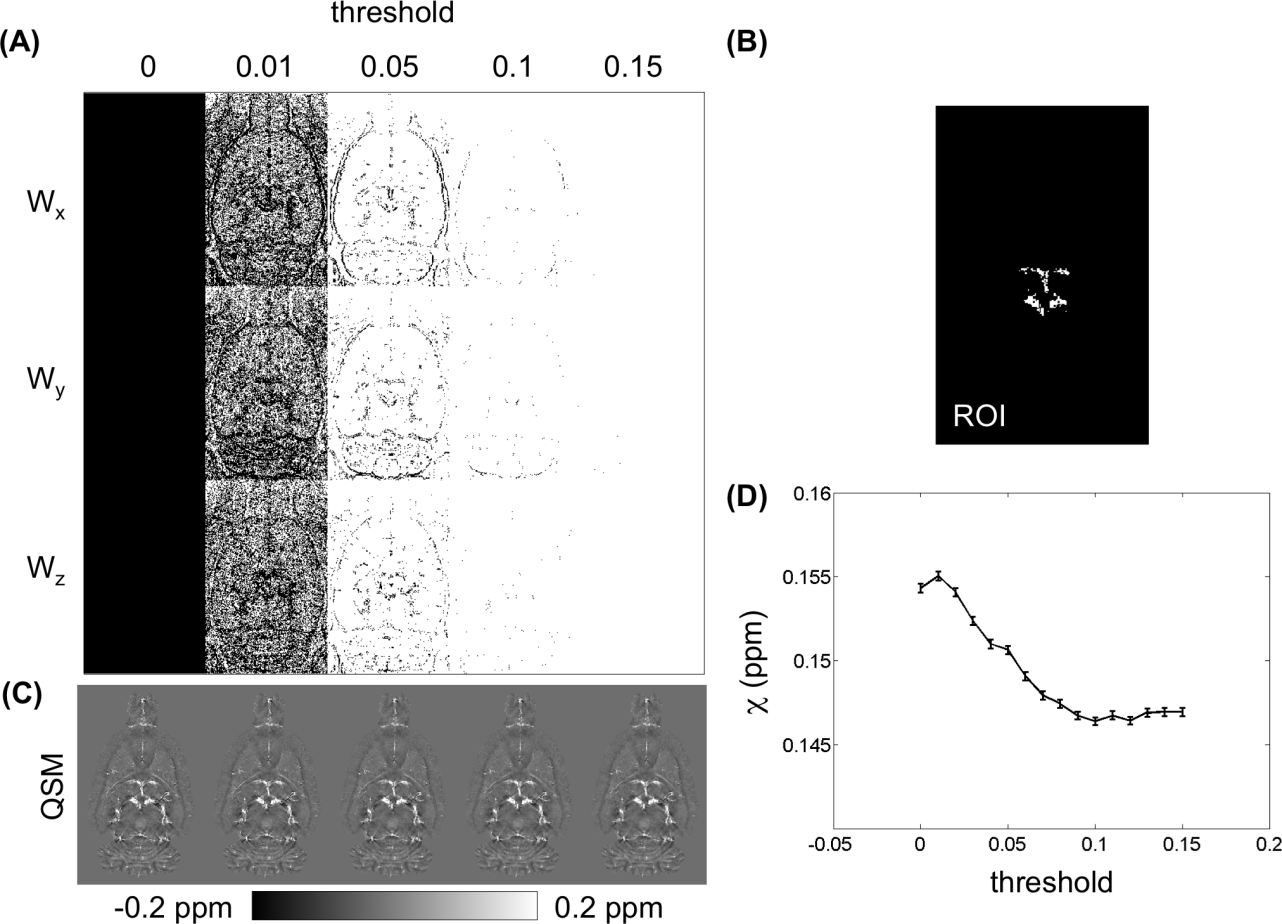
Influence of the selection of the prior information W. (A) Illustrations of the binary weighting in three dimensions across different thresholds from 0 to 0.15 (W_x_: weighting factor in x dimension; W_y_: weighting factor in y dimension; W_z_: weighting factor in z dimension;). (C) The reconstructed QSMs across different thresholds. (D) The susceptibility values measured from the region-of-interest (B) are the same as in Fig 4(J).

One challenge of QSM reconstruction is to select an appropriate value of the Lagrange multiplier (λ) and the prior information. In conventional L1 regularization, λ controls the fidelity of the reconstructed QSM. A large λ enforces minimization of the L1 norm term, which eliminates noise. In contrast, a small λ enforces data fidelity at the cost of streaking artifacts. λ is usually determined according to the L-curve criterion for the optimal QSM [48]. However, L1 regularization is usually underestimated [21,23,26,56]. In the present study, the magnitude prior L1 approach allows us to simultaneously suppress streaking artifacts and to prevent an over-smoothing QSM. In addition, the chosen prior information also influences the accuracy in reconstructed QSM [23,34,35]. Herein, the estimated susceptibility values were measured by varying the threshold of the magnitude gradient from 0 to 1 in the step of 0.01 (Fig 10). There were no significant differences between these QSMs (Fig 10C). However, the measured susceptibility declined with a larger threshold value (Fig 10D).

We found that, at baseline, the SvO_2_ in several major veins (MIF, LHIV, MCOLV, THSV, GCV, and STS) had a mean of 86.23%, which was higher than other SvO_2_ measurements of ~70% [17,58]. The overestimation of SvO_2_ (i.e., underestimation of susceptibility value) may be caused by partial volume effects. To date, the weighted L1 regularization was the most appropriate method of QSM reconstruction [1]. However, the partial volume effect resulted in an 18.6–33.8% underestimation of susceptibility (i.e., 10–16.6% relative overestimation of SvO_2_) according to our simulation (Table A in S3 File). Most of the quantified susceptibility values (~0.2 ppm) were about 33% lower than the true value (0.3 ppm) (Table A in S3 File). Thus, a correction factor of approximately 1.5 could be obtained for adjusting the SvO_2_ quantification in this study. Note that the quantified values depend on the size and the geometry of object, as well as TE. These critical factors should be considered carefully for future applications. Table 1 shows the 1-SvO_2_ ranged from roughly 10% to 18%. If the 1-SvO_2_ multiplies a correct factor of 1.5, the resultant 1-SvO_2_ were about 15–27%, which lead to oxygen level of 73–85%. This result after multiplying a correction factor is close to the SvO_2_ of ~70% reported by previous works [17,58]. The error due to partial volume effect could be improved by a recent method [59].

Furthermore, SvO_2_ estimates were calculated based on the association between the susceptibility differences of fully oxygenated and fully deoxygenated blood (Δχ_do_) (Eq 4). We used Δχ_do_ = 0.18 ppm (cgs), which has been used in other studies [11,13,37,60]. Conversely, Δχ_do_ = 0.27 ppm (cgs) was reported by Spees et al. [61], which has been used as the SvO_2_ value in other human brain experiments [62,63]. When Δχ_do_ = 0.27 ppm (cgs) was used in our analysis, the SvO_2_ results were from 88% to 93%, which are much higher than the normal SvO_2_ observed using MRI [17,58]. In addition, the quantified susceptibility value in this work was about 0.15 ppm as shown in Figs 3–5. After multiplying a correction factor of 1.5 as above mention, the resultant susceptibility was 0.22 ppm. If the Δχ_do_ = 0.27 ppm (cgs) was used, the resultant SvO_2_ become ~84%, which was in agreement with the values (i.e., 73–85%) discussed previously. Another error of this method is the choice of hematocrit value. We always assumed that Hct = 0.4 [38] in the present study. However, hematocrit varies between individuals (0.35–0.5) [13] and also depends upon vessel size [64].

QSM-mMRV was used to demonstrate its ability to longitudinally monitor the rehabilitation of a post-stroke rat. At 3 days after reperfusion, the SvO_2_ value of the ipsilateral cortex had significantly declined relative to the contralateral cortex, which agreed with a prior study [29]. At 7 and 10 days post reperfusion, the SvO_2_ value of ipsilateral cortex had gradually risen to the level of the contralateral cortex (*p* > 0.05), which indicated that the physiology of the post-stroke rat brain had almost recovered. These results are similar to those of a study [65] that used MRI to quantitatively observe the angiogenesis of a post-stroke rat brain. Moreover, we compared our SvO_2_ results with SpO_2_ measurements. Both of the blood oxygen values had gradually climbed after reperfusion. Relative to pulse oximeter, QSM-mMRV provided high spatial resolution and better penetration depth when measuring blood oxygen. In addition, we also found, in immunohistochemistry (IHC) staining of vessel cells, that the angiogenesis of the ipsilateral cortex area was greater 7 and 10 days after reperfusion (image not shown), and that the tendency of variation in angiogenesis was consistent with the QSM-mMRV and SvO_2_ measurements. Thus, we concluded that QSM-mMRV showed promise as a potential noninvasive observation tool for clinical applications.

The benefit of QSM-mMRV is using an intrinsic contrast agent (e.g., deoxy-hemoglobin) to detect the structural and quantitative information of venous vessels, and it is useful for longitudinal studies of vascular disease models. Many studies have investigated the microvascular structure and function using MRA methods: time-of-flight (TOF)-, phase-contrast (PC)-, ΔR_2_-, and ΔR_2_*-MRA [5,66–68]. TOF-MRA is widely used to visualize major arteries [5]. PC-MRA, based on calculating the phase shift, enables the visualization of arteries and veins [66]. Although both TOF- and PC-MRA provide structural and flow information, their ability to visualize microvessels is limited. ΔR_2_-MRA has been proposed to detect microvascular arterioles and venules and obtain cerebral blood volume (CBV) [67]. Nevertheless, iron-based contrast agents are problematic because of their availability, cost, and safety. Recent methods based on ΔR_2_*-MRA use blood oxygen-level-dependent (BOLD) contrast to detect venules and regional CBV [68]. Nonetheless, by acquiring two sets of 3D-GRE images under different inhalation conditions, ΔR_2_*-MRA is limited by its lengthy scan time (~76-min for an MR scan with two inhalation conditions) and difficult to use clinically.

QSM-mMRV simultaneously depicts vein architecture and provides quantitative information on SvO_2_. It does, however, have one limitation. QSM-mMRV based on a GRE sequence magnifies vessel size because of intravascular and extravascular dephasing. Park et al. [69] reported that the intracortical vessels (diameter: ≤ 80 μm) can be observed using 3D-GRE images. Ogawa and Lee [70] reported that the susceptibility effect caused the visual vessels to look twice their normal size in GRE images. This magnification in the magnitude of GRE images is caused by the extravascular dephasing component that depends on TE, field strength, vessel orientation, and voxel size. Relative to the magnitude of GRE images, recent studies on intracerebral microbleeds [71] and stroke [72] report that the visualized size from QSM is independent of TE.

## Conclusions

We have described a QSM-based microscopic MRV combined with QSM reconstruction for *in vivo* quantitative visualization of the architecture of small venous vessels in rat stroke model. The QSM corrects the nonlocal effects observed in SWI. Corrected by simulation results, the SvO_2_ estimated by QSM is ranged from 73% to 85% for healthy rats. The approach simultaneously offers cerebral *in vivo* microvascular structure and SvO_2_ measures, which can be used to evaluate the physiological and functional characteristics of microvascular changes over time. This technique might be further applied to monitor animal models or clinical patients with cerebrovascular disease.

## Supporting Information

S1 File QSM reconstruction. Figure A. Combined images from multichannel magnetic resonance data. Figure B. L-curve for magnitude prior L1-regularized quantitative susceptibility map (QSM) for a rat brain. Figure C. L-curve for L1-regularized quantitative susceptibility map (QSM) for an animal.
(PDF)

S2 File Comparison of the accuracy of the local fields acquired by path-based and Laplacian-based phase unwrapping was evaluated using numerical simulation. Figure A. Scheme of generating a simulation model. Figure B. Illustrations of the simulated field maps. Figure C. Comparison of the SHARP filtering method for Path- and Laplacian-based phase uwrapping algorithms.
(PDF)

S3 File Systematic error of the choice of the QSM method was estimated using a numerical simulation. Figures A–E. No partial volume effect (perpendicular to B_0_). Figures F–J. No partial volume effect (parallel to B_0_). Figures K–O. With partial volume effect (perpendicular to B_0_). Figures P–T. With partial volume effect (parallel to B_0_). Table A. Quantified results from simulation images at three different echo times and two orientations.
(PDF)
