# Supplementary material for: Quantitative Susceptibility Mapping-Based Microscopy of Magnetic Resonance Venography (QSM-mMRV) for In Vivo Morphologically and Functionally Assessing Cerebromicrovasculature in Rat Stroke Model
Source: PLoS One. 2016 Mar 14;11(3):e0149602. doi: 10.1371/journal.pone.0149602 (PMC4790912; doi:10.1371/journal.pone.0149602)
Supplement: S1 File — Figure A. Combined images from multichannel magnetic resonance data. Figure B. L-curve for magnitude prior L1-regularized quantitative susceptibility map (QSM) for a rat brain. Figure C. L-curve for L1-regularized quantitative susceptibility map (QSM) for an animal. (PDF) [file pone.0149602.s001.pdf]

Quantitative Susceptibility Mapping-Based Microscopy of Magnetic Resonance Venography (QSM-mMRV) for *In Vivo* Morphologically and Functionally Assessing Cerebromicrovasculature in Rat Stroke Model

(S1 Supporting Information)

QSM reconstruction

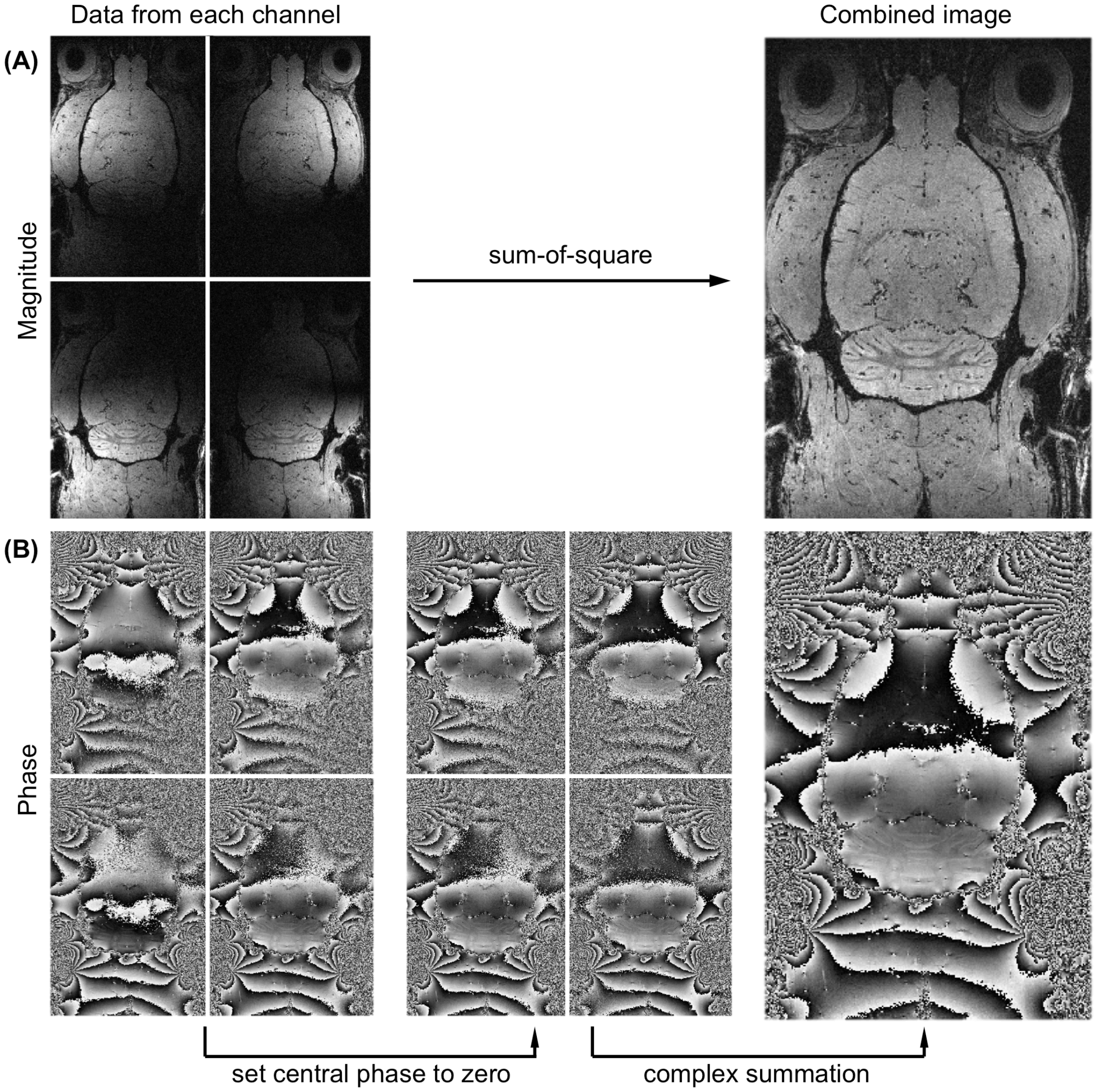

Figure A. Combined images from multichannel magnetic resonance data. (A) Magnitude images were combined using sum-of-squares. (B) Each phase image first had its central phase set to zero and was then combined using complex summation.

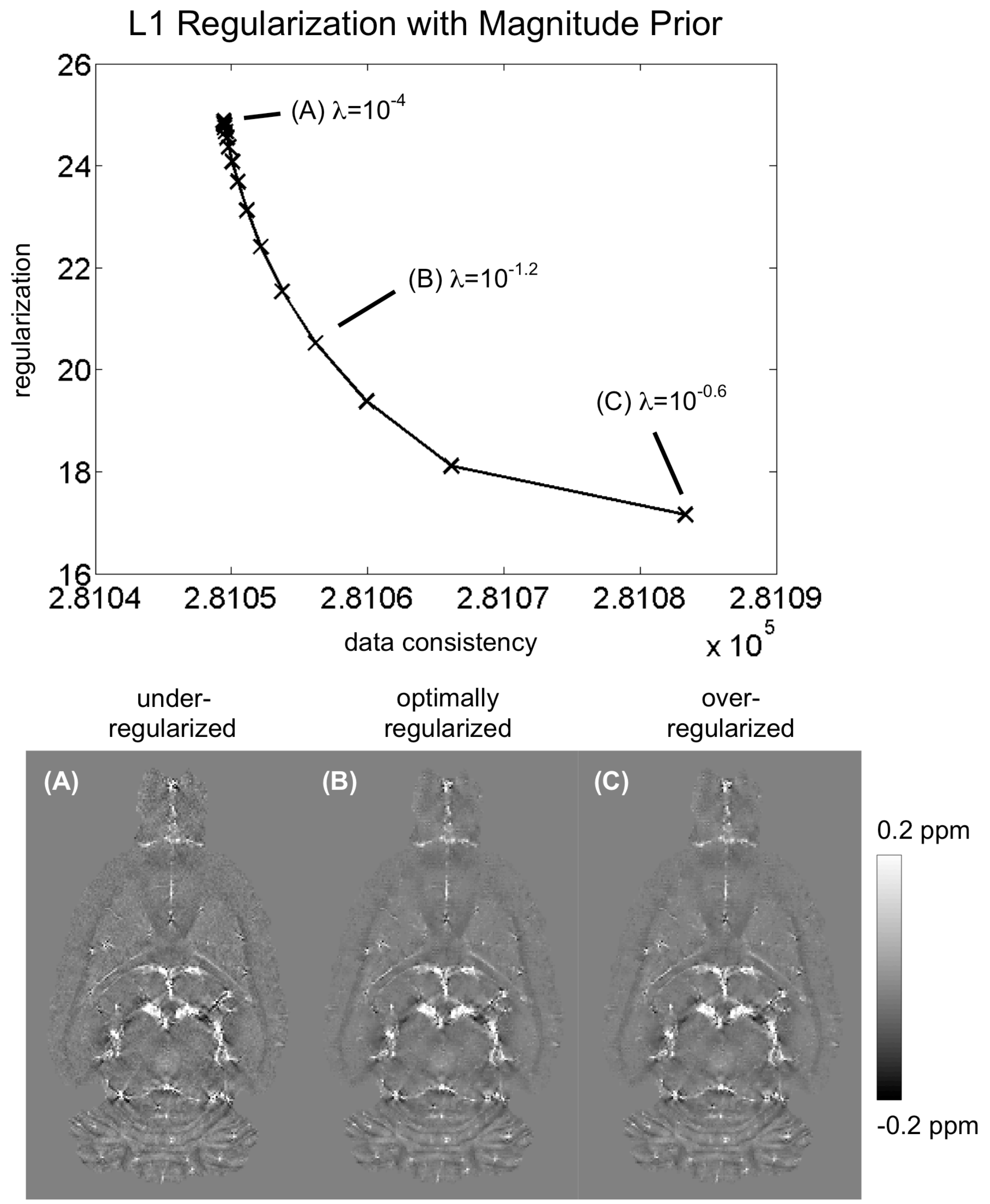

Figure B. L-curve for magnitude prior L1-regularized quantitative susceptibility map (QSM) for a rat brain. X-axis: data consistency term $\frac{1}{2}\left\|b - F^{-1}DF\chi\right\|_2^2$ in regularized reconstruction for the varying value of the Lagrange parameter λ. Y-axis: regularization term $\left\|WG\chi\right\|_1$. (A) Setting $\lambda = 10^{-4}$ yielded an under-regularized QSM. (B) For $\lambda = 10^{-1.2}$, the operating point with the largest curvature on the L-curve was obtained. (C) Setting $\lambda = 10^{-0.6}$ yielded an over-regularized QSM.

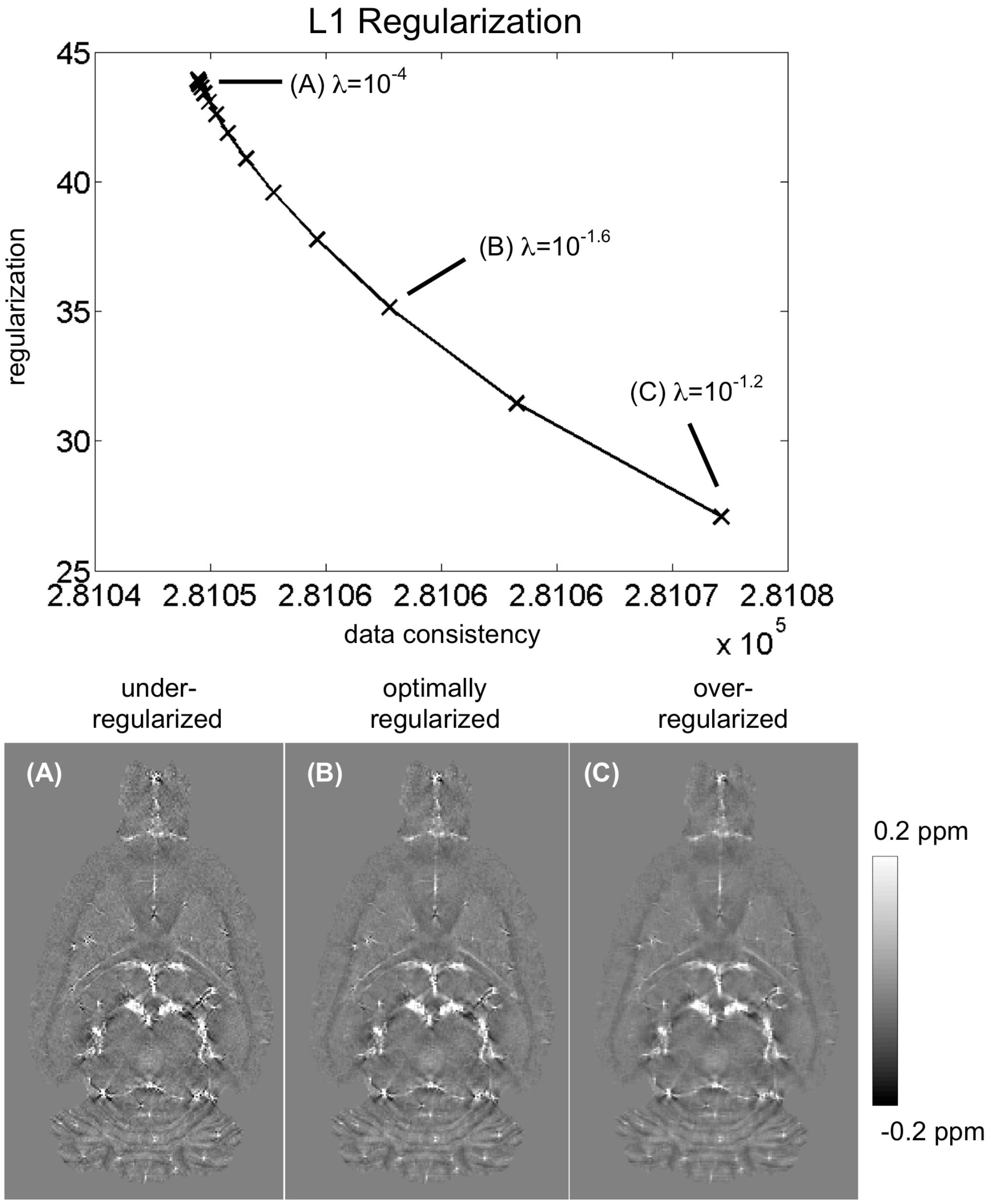

Figure C. L-curve for L1-regularized quantitative susceptibility map (QSM) for an animal. X-axis: data consistency term $\frac{1}{2}\left\|b - F^{-1}DF\chi\right\|_2^2$ in regularized reconstruction for the varying value of the Lagrange parameter λ. Y-axis: regularization term $\left\|G\chi\right\|_1$. (A) Setting $\lambda = 10^{-4}$ yielded an under-regularized QSM. (B) For $\lambda = 10^{-1.6}$, the operating point with the largest curvature on the L-curve was obtained. (C) Setting $\lambda = 10^{-1.2}$ yielded an over-regularized QSM.
