# Supplementary material for: Quantitative Susceptibility Mapping-Based Microscopy of Magnetic Resonance Venography (QSM-mMRV) for In Vivo Morphologically and Functionally Assessing Cerebromicrovasculature in Rat Stroke Model
Source: PLoS One. 2016 Mar 14;11(3):e0149602. doi: 10.1371/journal.pone.0149602 (PMC4790912; doi:10.1371/journal.pone.0149602)
Supplement: S2 File — Figure A. Scheme of generating a simulation model. Figure B. Illustrations of the simulated field maps. Figure C. Comparison of the SHARP filtering method for Path- and Laplacian-based phase uwrapping algorithms. (PDF) [file pone.0149602.s002.pdf]

Quantitative Susceptibility Mapping-Based Microscopy of Magnetic Resonance Venography (QSM-mMRV) for *In Vivo* Morphologically and Functionally Assessing Cerebromicrovasculature in Rat Stroke Model

(S2 Supporting Information)

Comparison of the accuracy of the local fields acquired by the path-based [1] and Laplacian-based [2] phase unwrapping was evaluated using numerical simulation.

Methods

The scheme of generating a simulation model was shown in Fig. A. Field of view (FOV): 3.2 × 3.2 × 3.2 mm^3, matrix size (MTX): 512 × 512 × 512. The simulated model was composed of three parts: a vessel (cylinder), a parenchyma (cube), and a source producing a background field (surrounding the parenchyma). A cylinder (radius: 32 voxels; height: 224 voxels; offset from center: 5 voxels) with a susceptibility of 0.3 ppm was created. The susceptibility of the parenchyma (reference of susceptibility) was set at 0 ppm. The background field was generated from the source (susceptibility value: 3 ppm) outside the water box (matrix size: 448 × 448 × 448) (Fig. BC). This susceptibility value was chosen because it resulted in background field perturbations with a strength similar to that usually observed in real phantom experiments with water and air as the surrounding media, although the susceptibility difference is only 1/3 of that between water and air (approximately −9 ppm). This discrepancy might be attributable to a lack of shimming fields in the simulation. The total field was generated by convolving the point-spread function in a Fourier domain [3] (Fig. BD). To simulate the partial volume and Gibb's ring effects, the subsampled image with a partial volume effect was obtained by reducing the image resolution from 6.25 to 100 μm isotropic (reduction factor = 16). The zero mean Gaussian noise was separately added to the

real and imaginary parts of *k*-space (signal-to-noise ratio [SNR] of magnitude = 50). The wrapped phase was obtained with the main magnet (7 Tesla), the gyromagnetic ratio of ^{1}H (42.58 MHz), and echo time of 10 ms.

Results

The unwrapped phase images were calculated by (Fig. CA) path-based and (Fig. CD) Laplacian-based unwrapping algorithms. The estimated local field image (Fig. CB) and (Fig. CE) were obtained using SHARP filtering [4] with the radius of 3 voxels (shell thickness was set as 1 voxel) from (Fig. CA) and (Fig. CD), respectively. The QSMs calculated from (Fig. CB) and (Fig. CE) are shown in Fig. CC and Fig. CF, respectively. The quantified susceptibility value were 0.22 and 0.19 ppm in Fig. CC and Fig. CF, respectively. The relative errors of estimated susceptibility from Fig. CC and Fig. CF were –25.91% and –35.43%, respectively. Using path-based phase unwrapping obtained less error in QSM relative to Laplacian-based unwrapping algorithm.

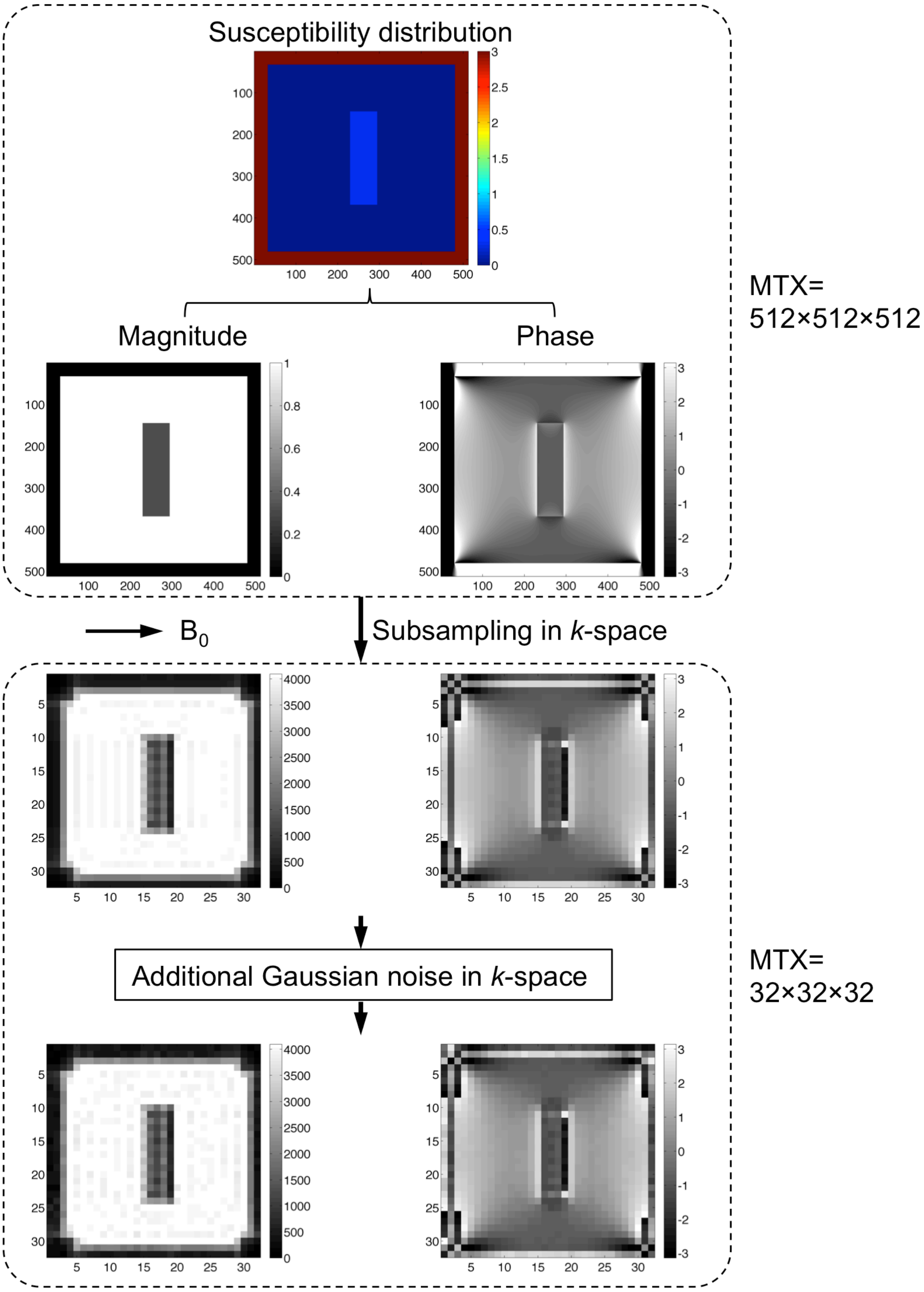

Figure A. Scheme of generating a simulation model.

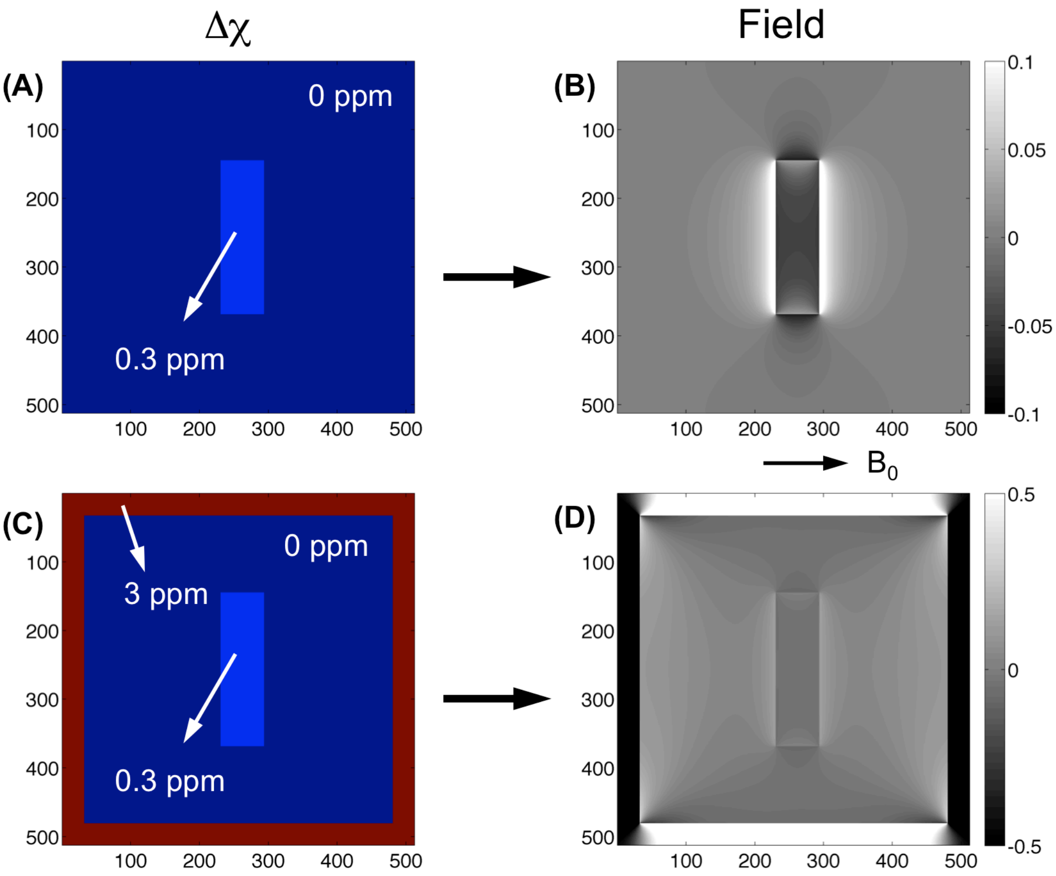

Figure B. Illustrations of the simulated field maps. (A) Susceptibility distribution without background source. (B) Field map without a background field generated from (A). (C) Susceptibility distribution with a background source of 3 ppm. (D) Field map with a background field generated from (C).

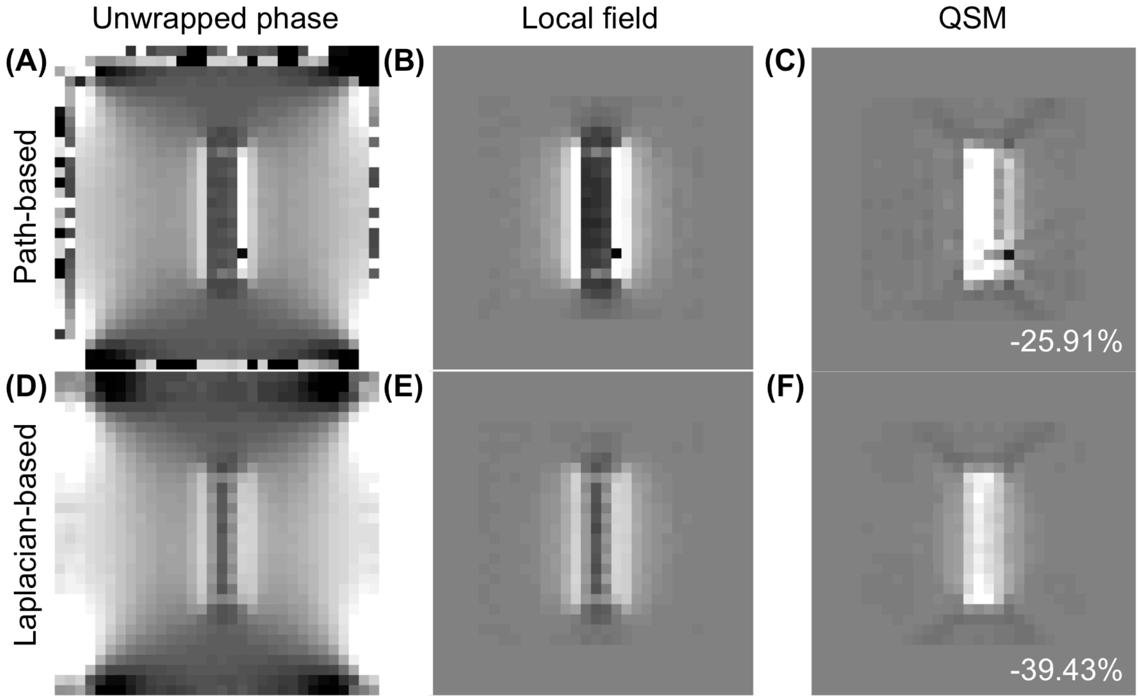

Figure C. Comparison of the SHARP filtering method for Path- and Laplacian-based phase uwrapping algorithms. (A) Path-based and (D) Laplacian-based unwrapped phase images. (B) Estimated local field by SHARP filtering from (A). (E) Estimated local field by SHARP filtering from (D). (C) and (F) were calculated from (B) and (E), respectively. The relative errors of estimated susceptibility of (C) and (F) were –25.91% and –35.43%, respectively.

References

1. Abdul-Rahamn HS, Gdeisat MA, Burton DR, Lalor MJ, Lilley F, et al. Fast and robust three-dimensional best path phase unwrapping algorithm. Appl Opt 2007;46:6623–6635.
2. Li W, Wu B, Liu C. Quantitative susceptibility mapping of human brain reflects spatial variation in tissue composition. Neuroimage 2011;55:1645–1656.
3. Salomir R, de Senneville BD, Moonen CT. A fast calculation method for magnetic field inhomogeneity due to an arbitrary distribution of bulk susceptibility. Concept Magn Res Part B 2003;19:26–34.
4. Schweser F, Deistung A, Lehr BW, Reichenbach JR. Quantitative imaging of intrinsic magnetic tissue properties using MRI signal phase: An approach to *in vivo* brain iron metabolism? Neuroimage 2011; 54:2789–2807.
