# Supplementary material for: Quantitative Susceptibility Mapping-Based Microscopy of Magnetic Resonance Venography (QSM-mMRV) for In Vivo Morphologically and Functionally Assessing Cerebromicrovasculature in Rat Stroke Model
Source: PLoS One. 2016 Mar 14;11(3):e0149602. doi: 10.1371/journal.pone.0149602 (PMC4790912; doi:10.1371/journal.pone.0149602)
Supplement: S3 File — Figures A–E. No partial volume effect (perpendicular to B0). Figures F–J. No partial volume effect (parallel to B0). Figures K–O. With partial volume effect (perpendicular to B0). Figures P–T. With partial volume effect (parallel to B0). Table A. Quantified results from simulation images at three different echo times and two orientations. (PDF) [file pone.0149602.s003.pdf]

Quantitative Susceptibility Mapping-Based Microscopy of Magnetic Resonance Venography (QSM-mMRV) for *In Vivo* Morphologically and Functionally Assessing Cerebromicrovasculature in Rat Stroke Model

(S3 Supporting Information)

Systematic error of the choice of the QSM method was estimated using a numerical simulation.

Methods

The simulation model is described in Supporting Information 2 (S2). We simulated objects at three echo times (TE=5, 10, 15 ms) and two orientations (parallel [angle = 0°] and perpendicular [angle = 90°] to B_0). The simulation models with and without a partial volume effect (PVE) were created as well.

Results

The QSM, quantified result, and relative error for each condition are shown in following figures. Table A summaries the quantified results while the optimal parameter were used. Under the condition of no partial volume effects, the quantified susceptibility values were ranged from 0.27 to 0.28 ppm, and the corresponding systematic errors were ranged from 5.5% to 8.8%. With the partial volume effects, the quantified susceptibility values were ranged from 0.2 to 0.24 ppm, and the corresponding systematic errors were ranged from 18.6% to 33.8%.

No Partial Volume Effect (perpendicular to B0)

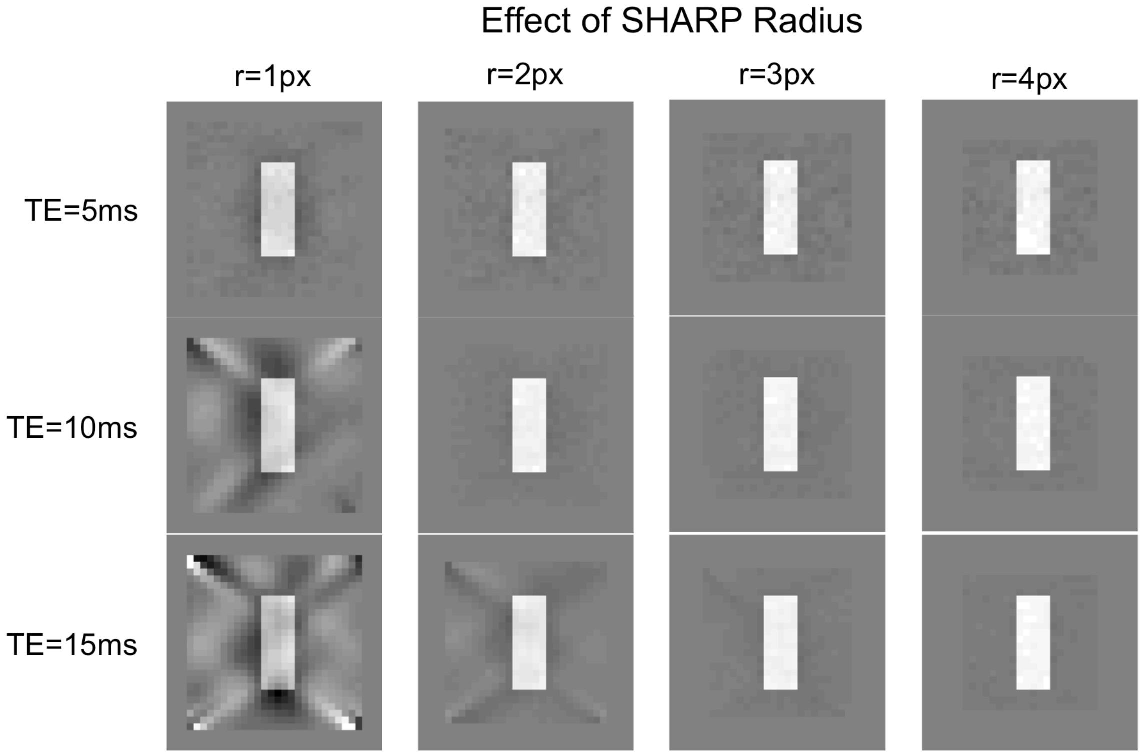

Figure A. QSMs using various SHARP radius at three different TEs. (λ=10^{-1}; threshold of W=0.1).

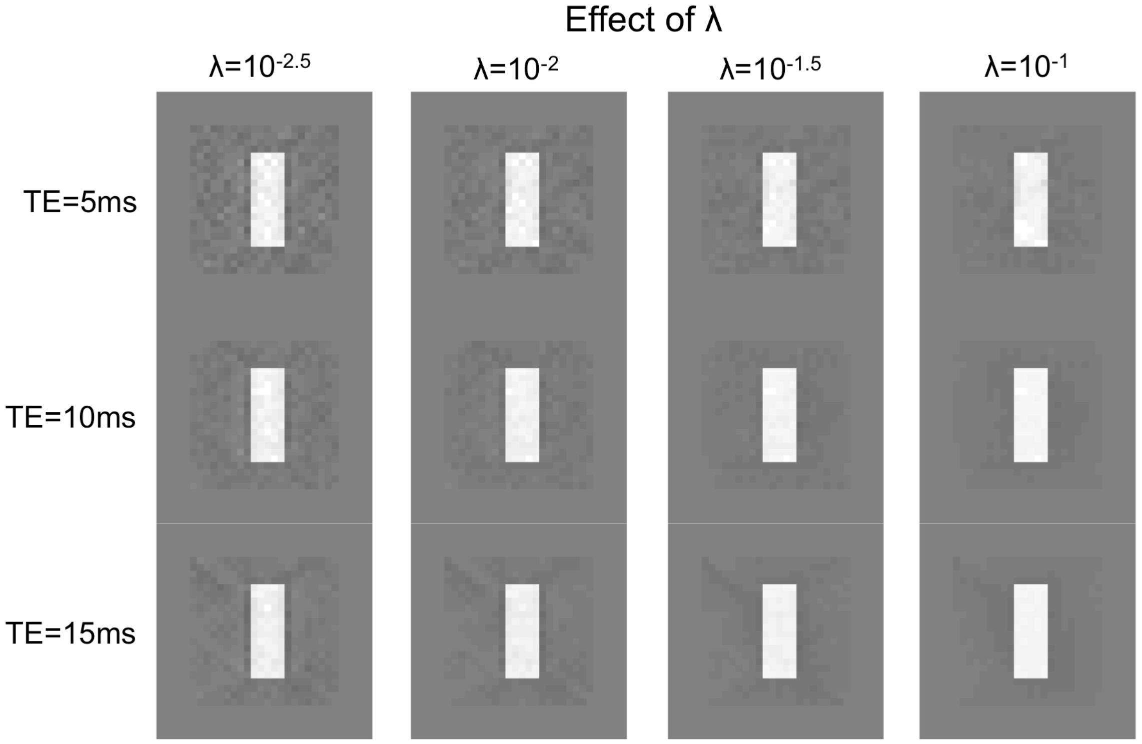

Figure B. QSMs using various λ at three different TEs. (SHARP radius=3 px; threshold

of W=0.1).

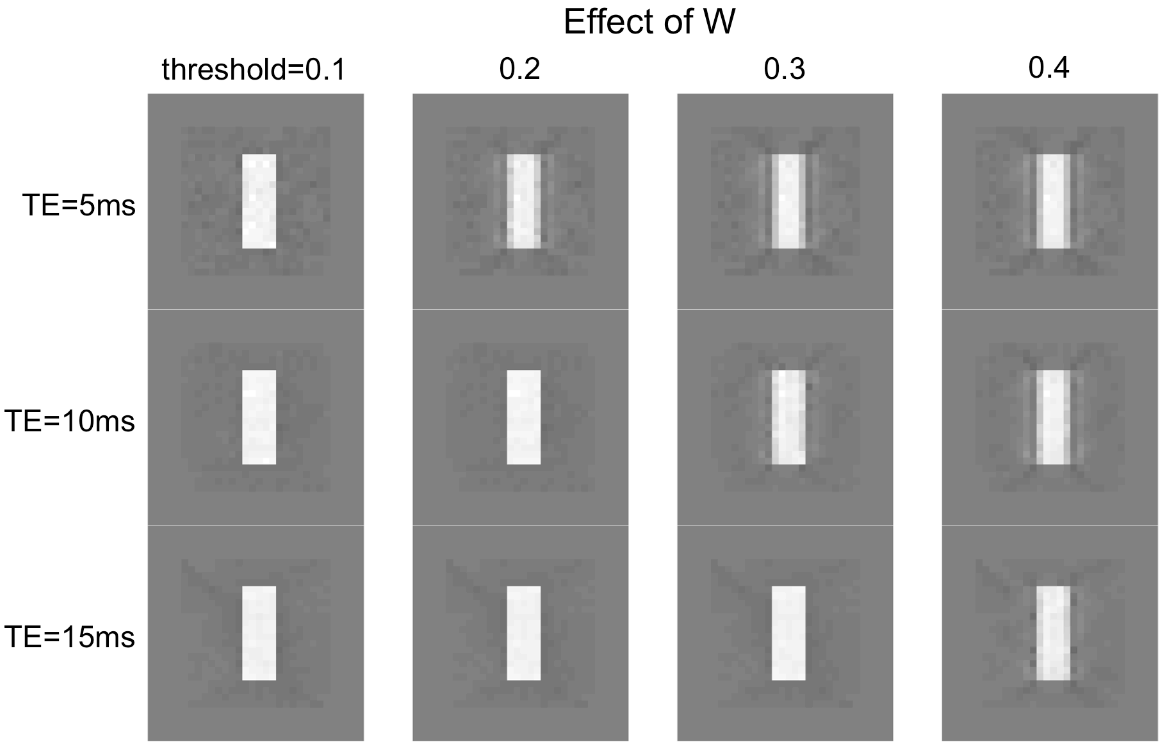

Figure C. QSMs using various thresholds of W at three different TEs. (SHARP radius=3 px; λ=10^{-1}).

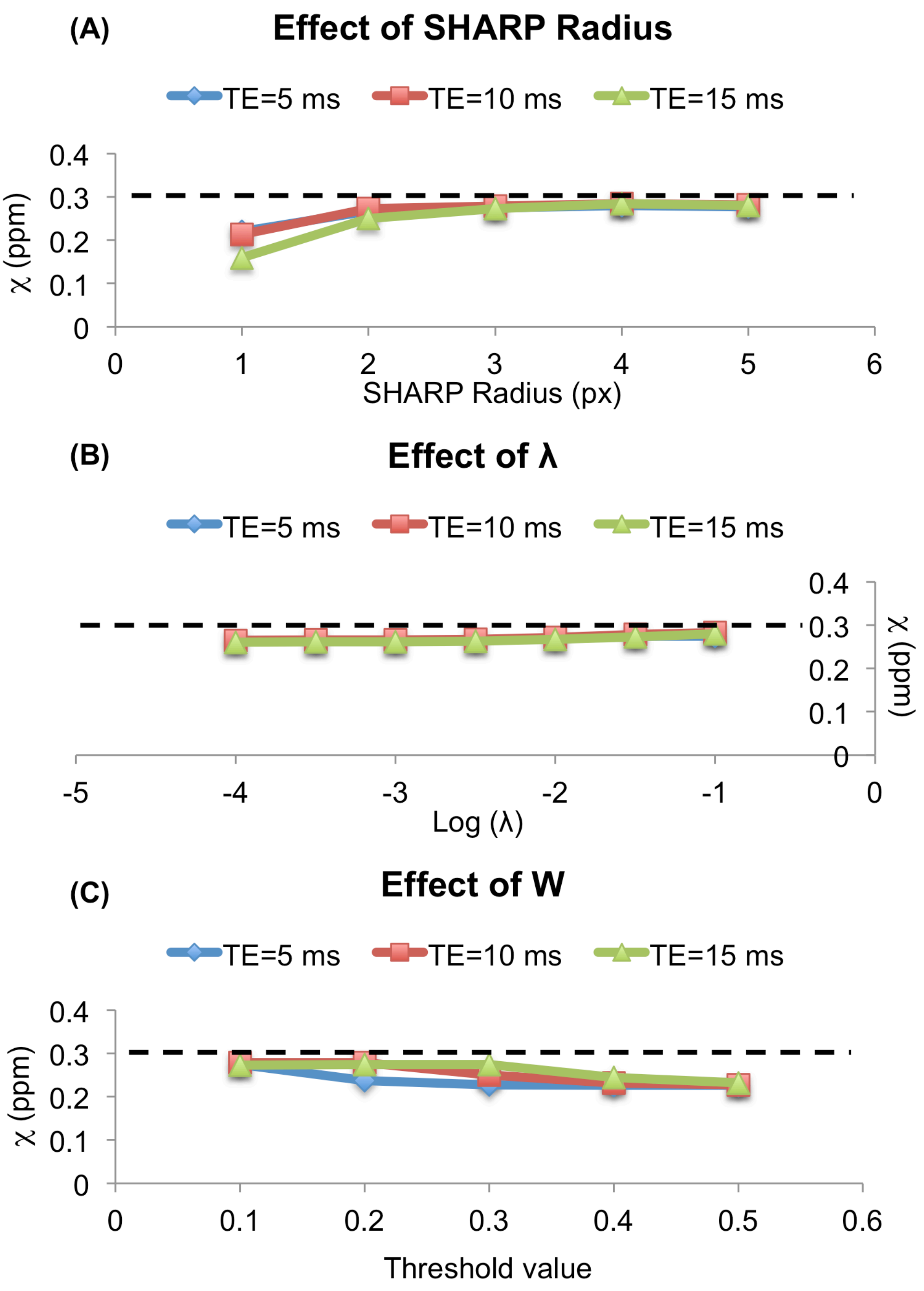

Figure D. Quantified results using various (A) SHARP radius (λ=10^{-1}; threshold of W=0.1), (B) λ (SHARP radius=3 px; threshold of W=0.1), and (C) threshold of W (SHARP radius=3 px; λ=10^{-1}).

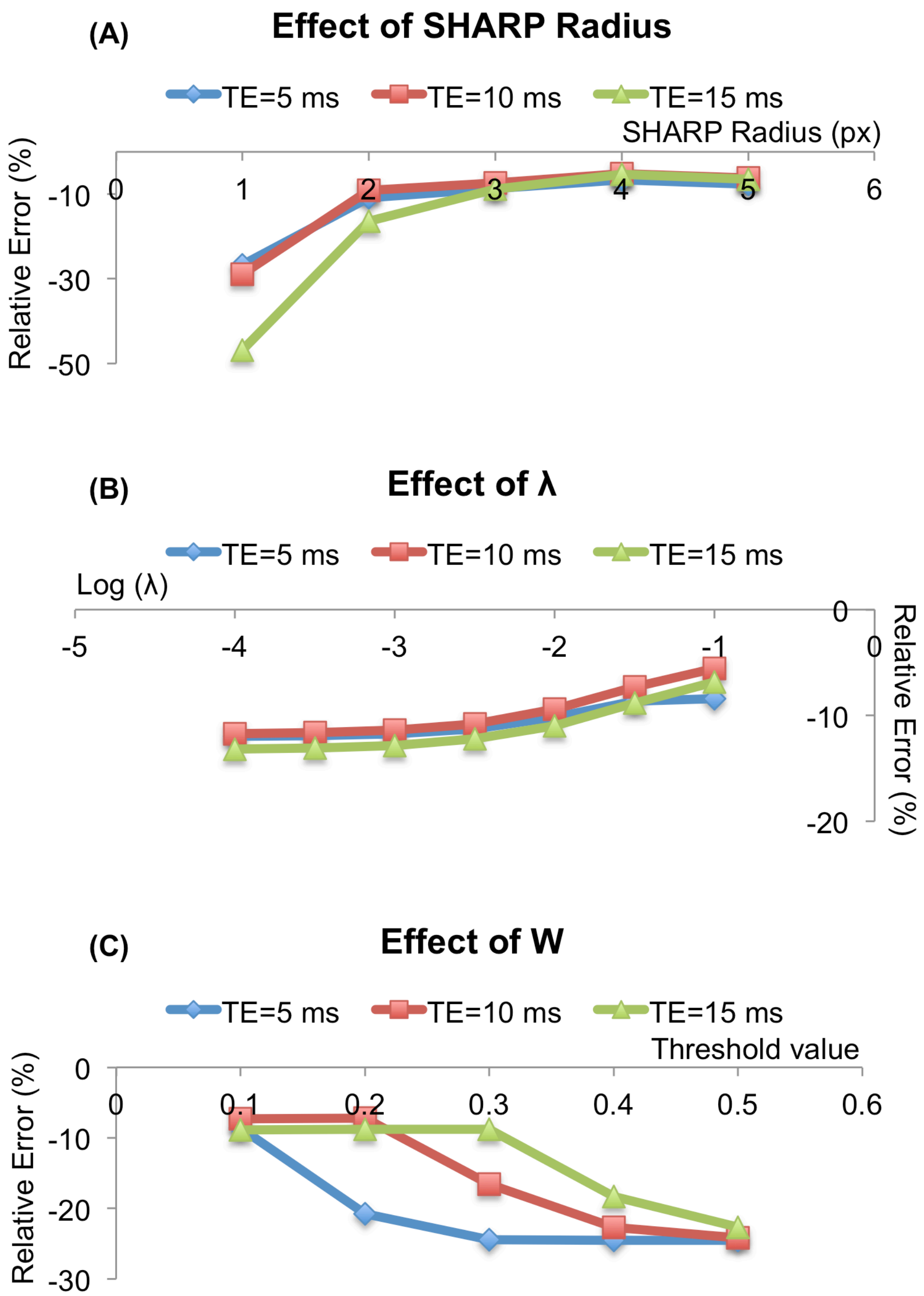

Figure E. Relative error using various (A) SHARP radius ($\lambda=10^{-1}$; threshold of W=0.1), (B) λ (SHARP radius=3 px; threshold of W=0.1), and (C) threshold of W (SHARP radius=3 px; $\lambda=10^{-1}$).

No Partial Volume Effect (parallel to B0)

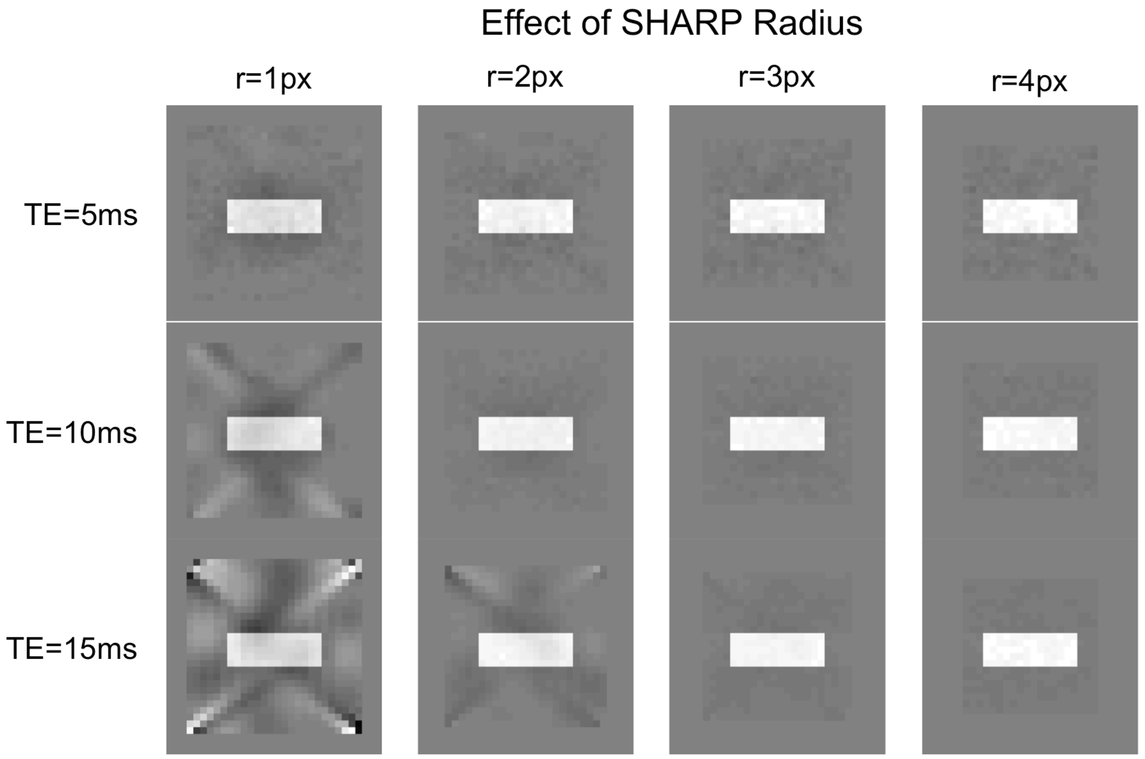

Figure F. QSMs using various SHARP radius at three different TEs. ($\lambda=10^{-1}$; threshold of W=0.1).

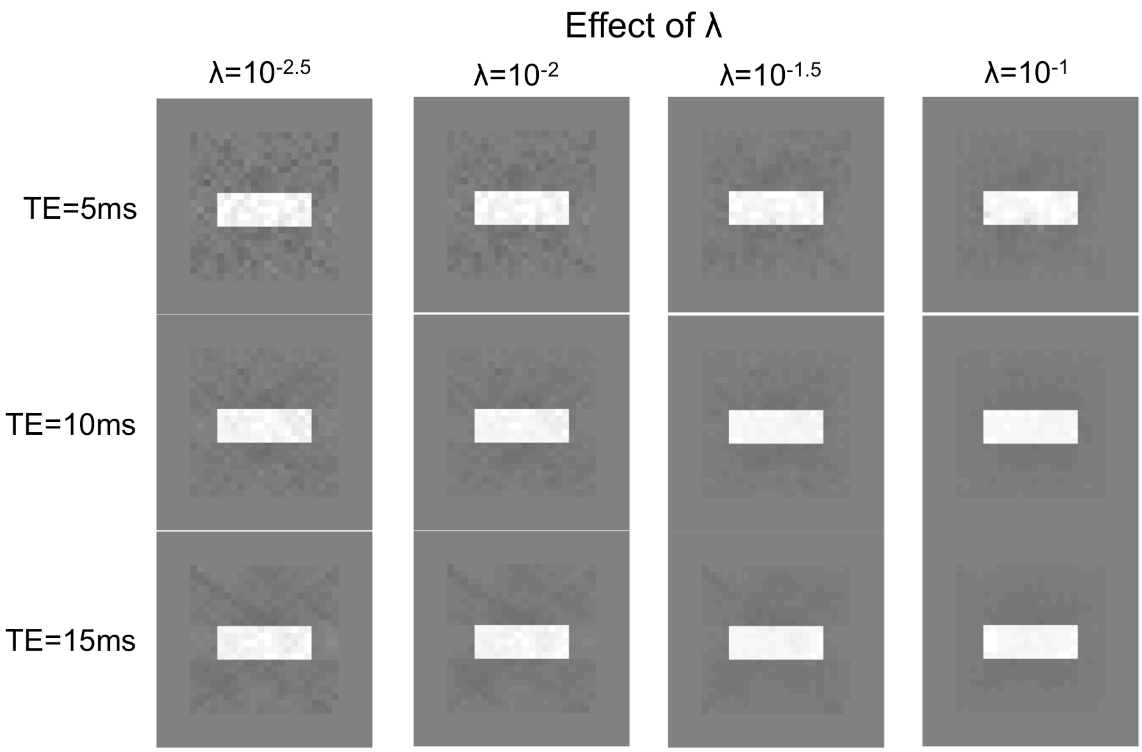

Figure G. QSMs using various λ at three different TEs. (SHARP radius=3 px; threshold of W=0.1).

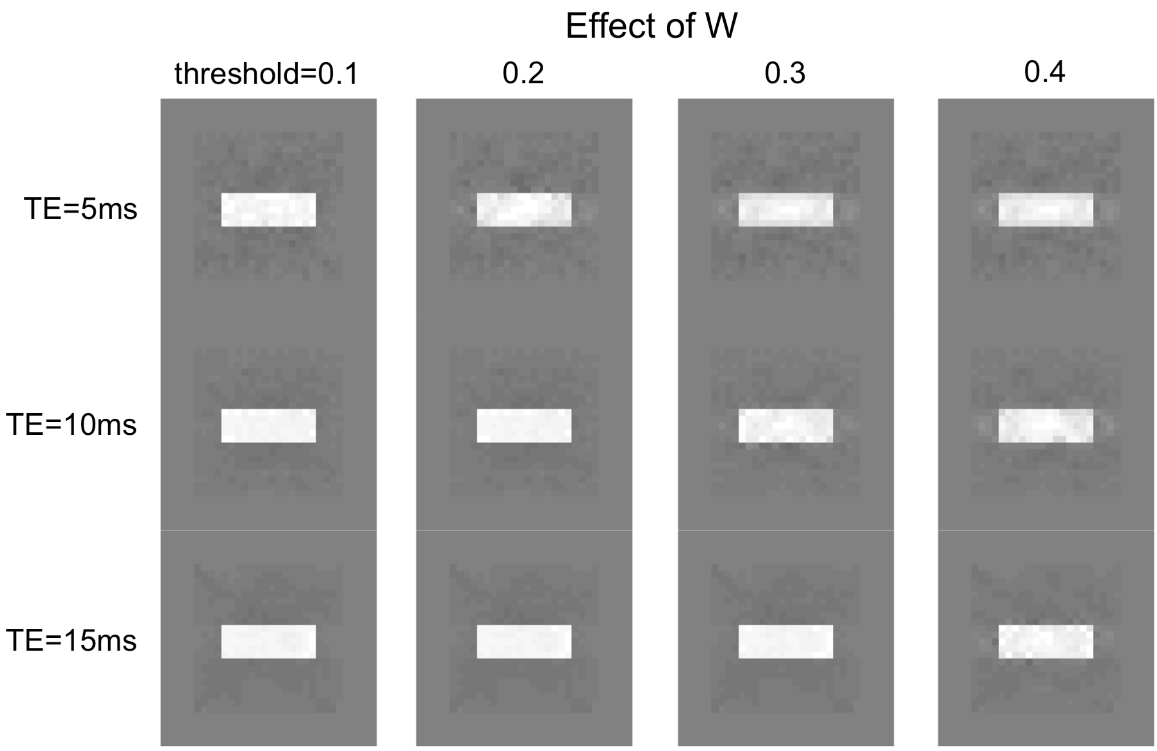

Figure H. QSMs using various thresholds of W at three different TEs. (SHARP

radius=3 px; λ=10^{-1}).

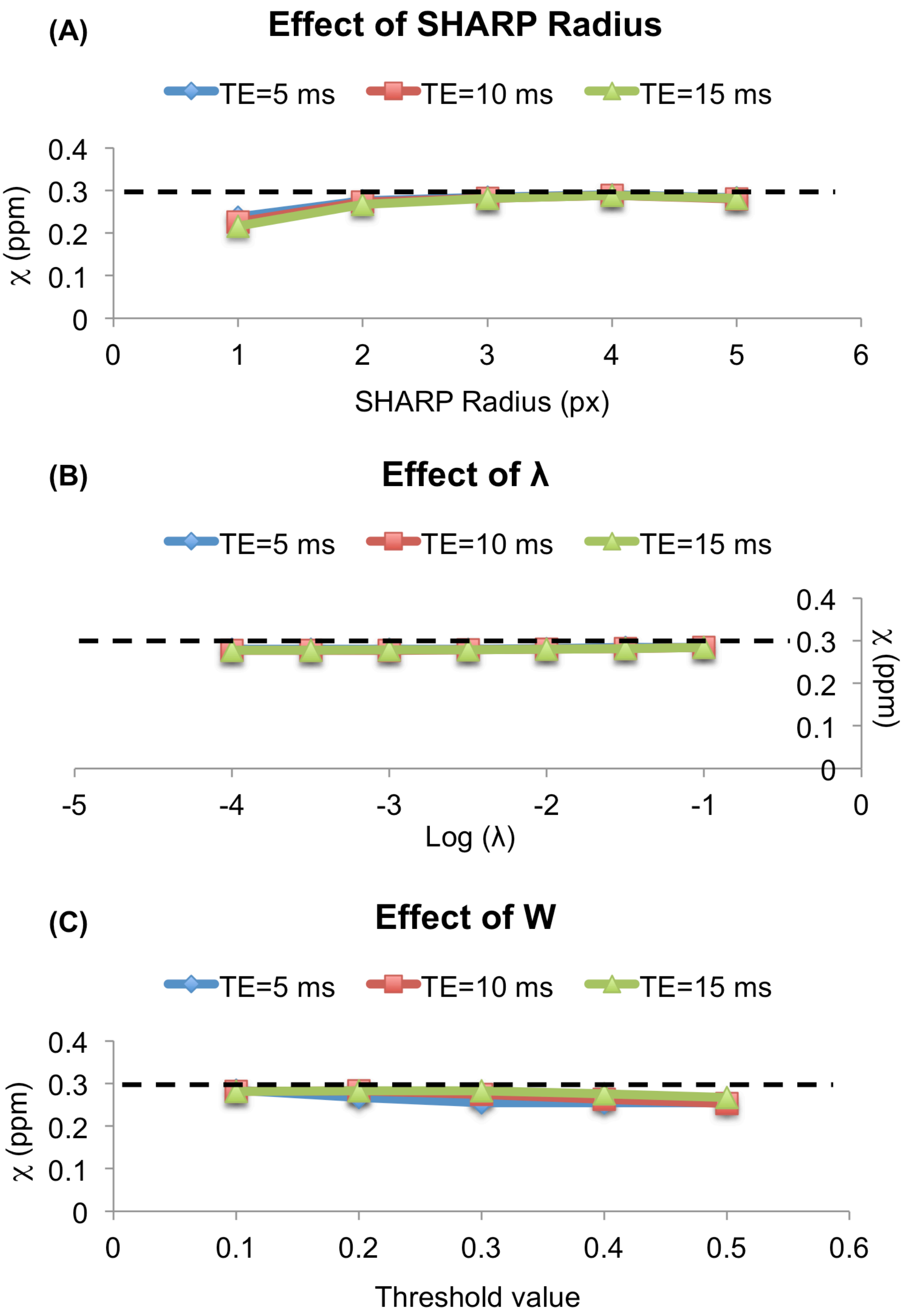

Figure I. Quantified results using various (A) SHARP radius (λ=10^{-1}; threshold of W=0.1), (B) λ (SHARP radius=3 px; threshold of W=0.1), and (C) threshold of W (SHARP radius=3 px; λ=10^{-1}).

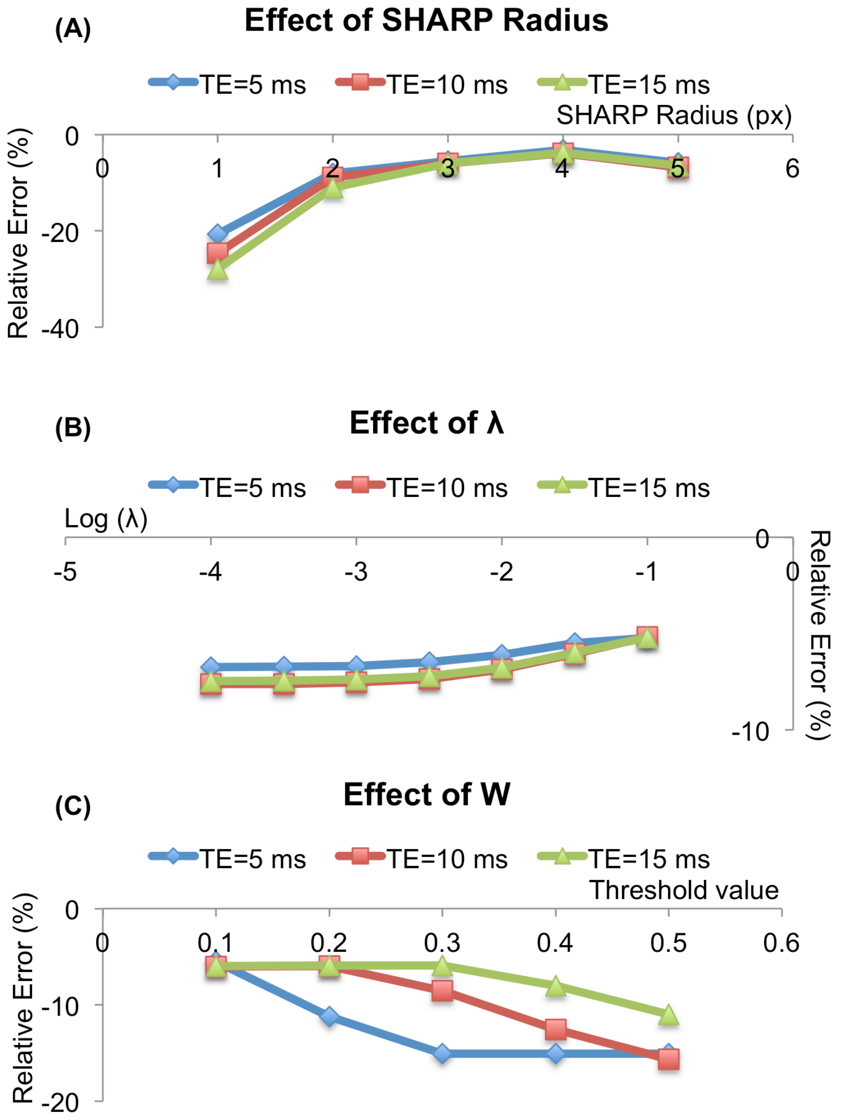

Figure J. Relative error using various (A) SHARP radius ($\lambda=10^{-1}$; threshold of W=0.1), (B) λ (SHARP radius=3 px; threshold of W=0.1), and (C) threshold of W (SHARP radius=3 px; $\lambda=10^{-1}$).

With Partial Volume Effect (perpendicular to B0)

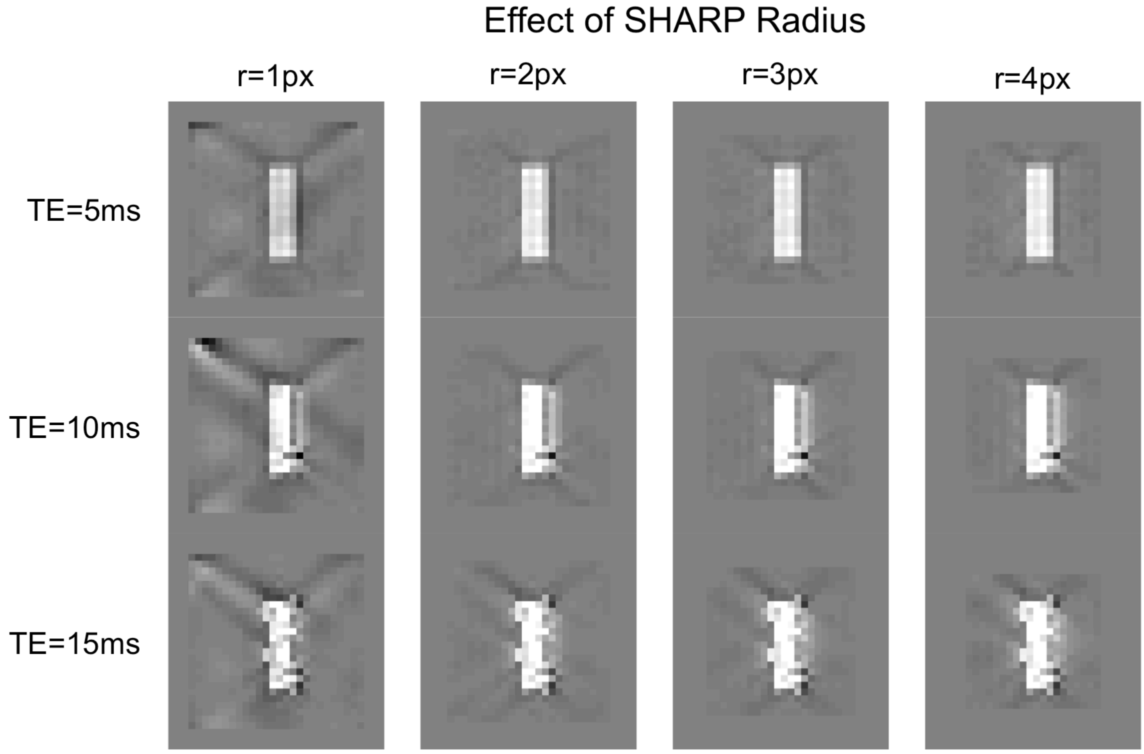

Figure K. QSMs using various SHARP radius at three different TEs. (λ=$10^{-1.5}$; threshold of W=0.3).

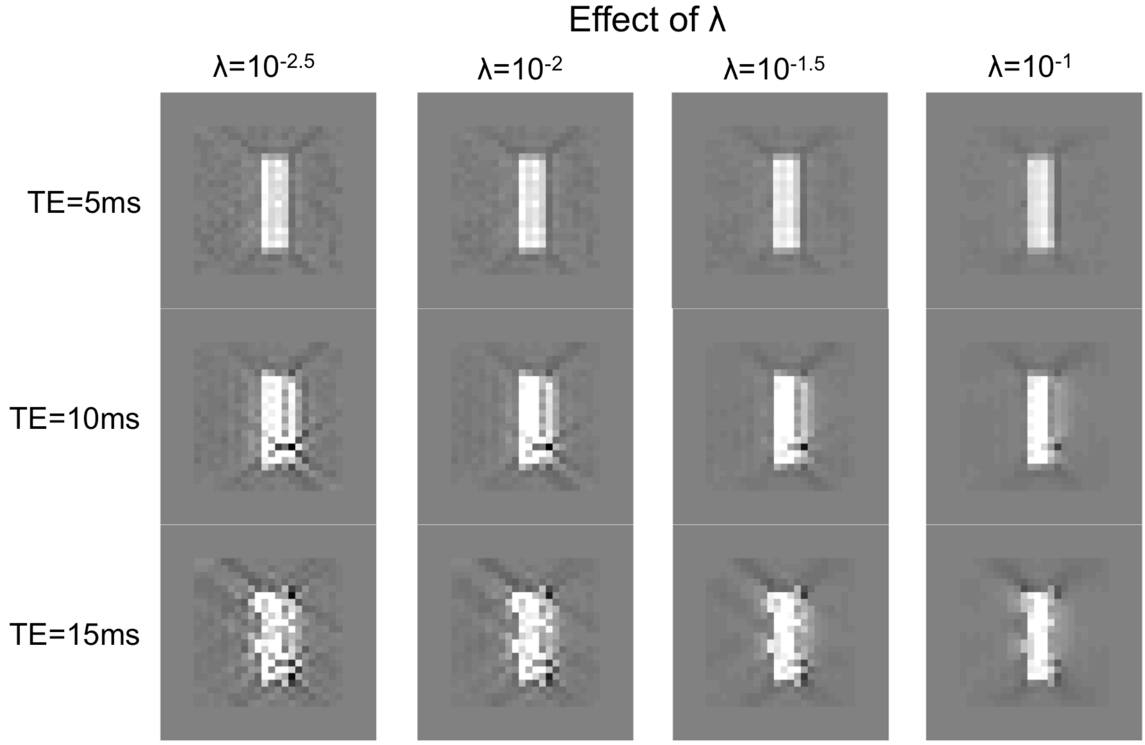

Figure L. QSMs using various λ at three different TEs. (SHARP radius=3 px; threshold of W=0.3).

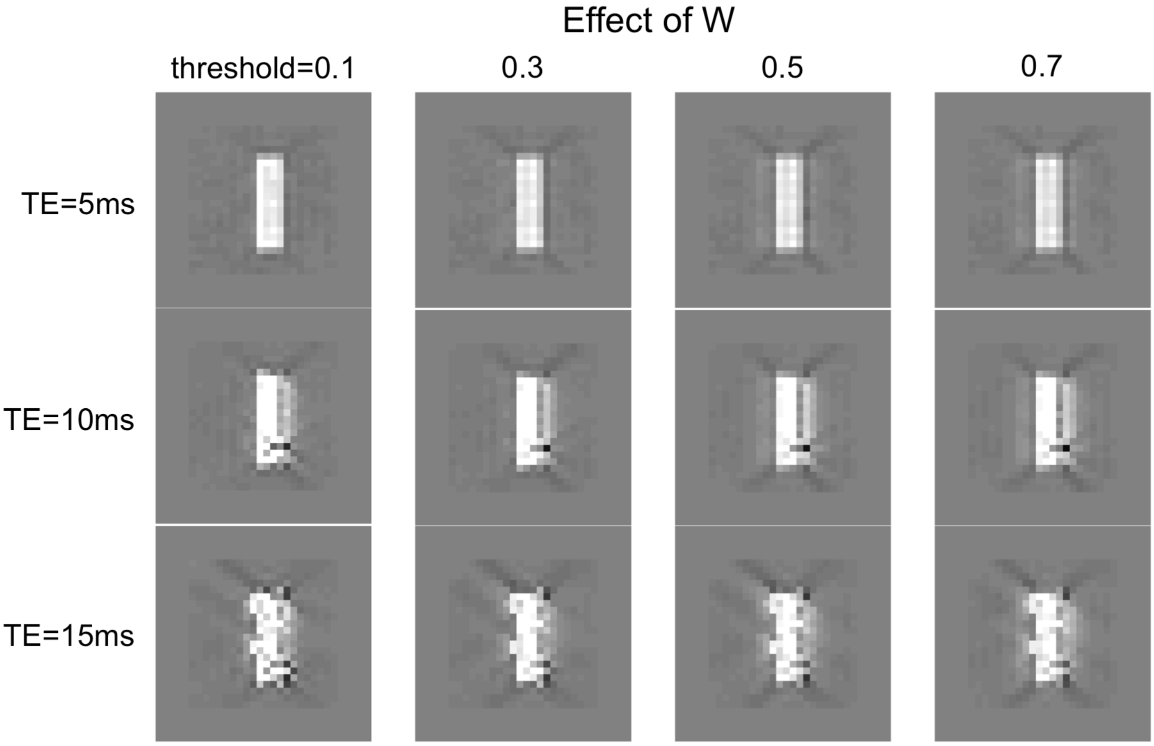

Figure M. QSMs using various thresholds of W at three different TEs. (SHARP radius=3 px; $\lambda=10^{-1.5}$).

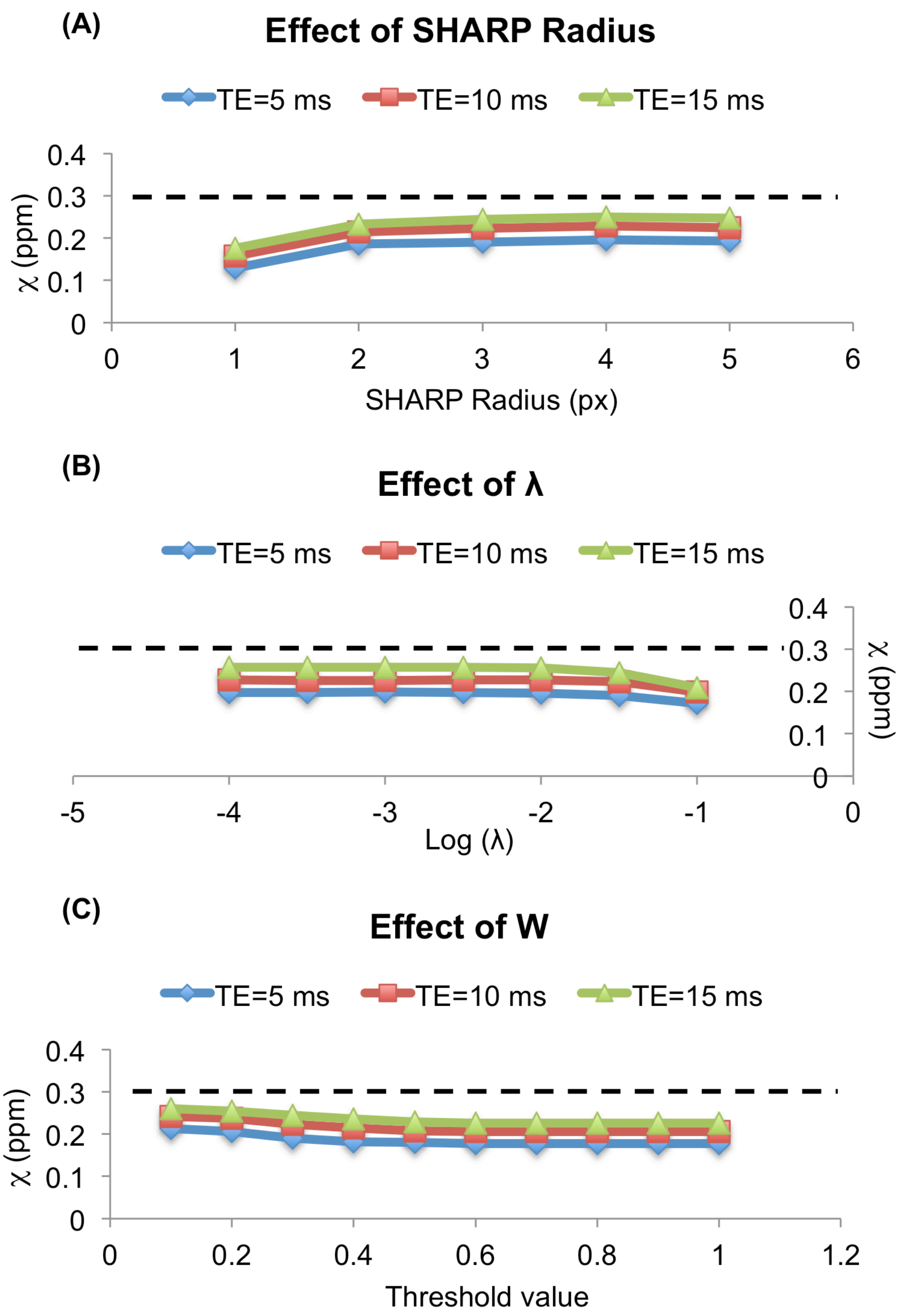

Figure N. Quantified results using various (A) SHARP radius ($\lambda=10^{-1.5}$; threshold of W=0.3), (B) λ (SHARP radius=3 px; threshold of W=0.3), and (C) threshold of W (SHARP radius=3 px; $\lambda=10^{-1.5}$).

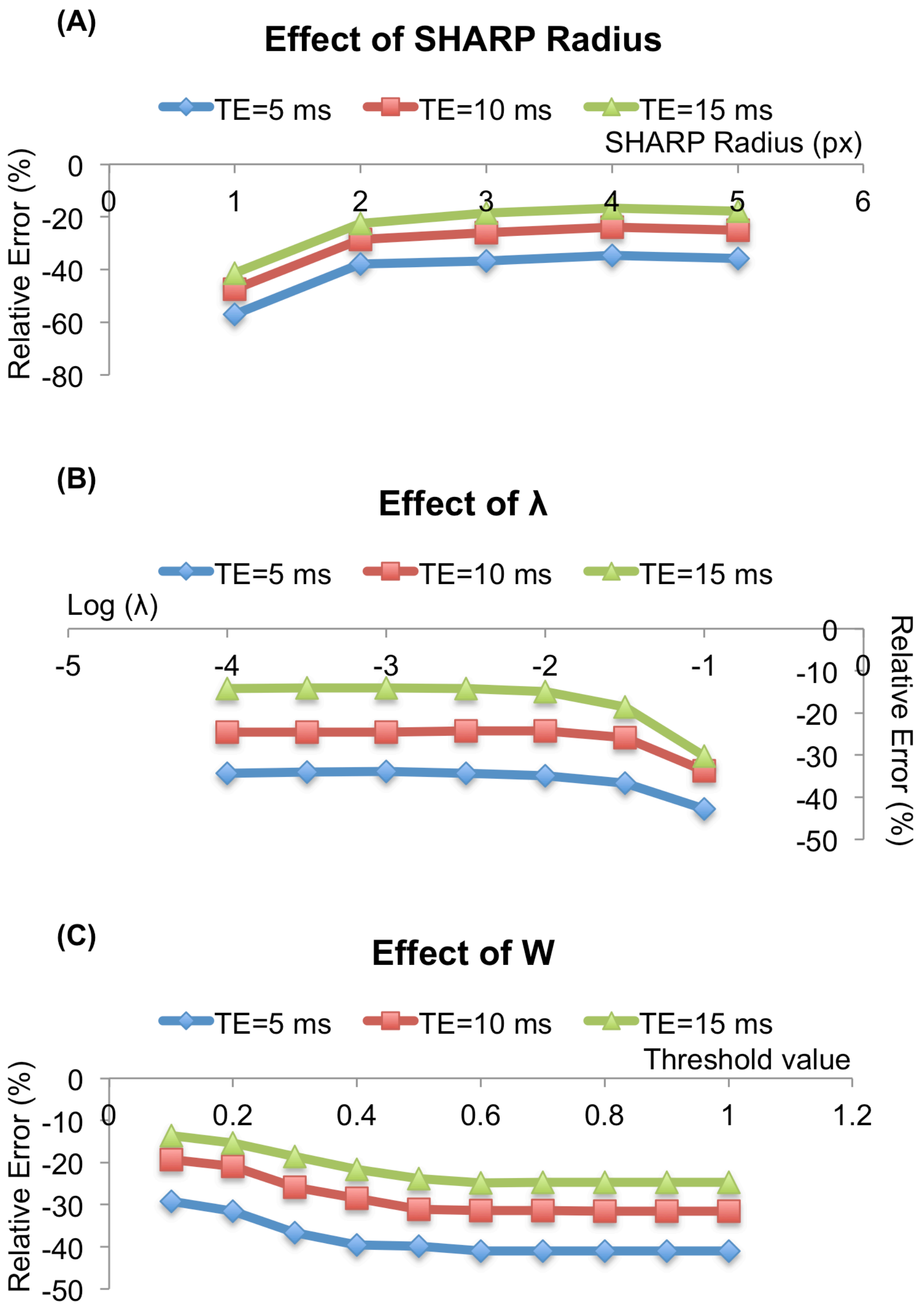

Figure O. Relative error using various (A) SHARP radius ($\lambda=10^{-1.5}$; threshold of W=0.3), (B) λ (SHARP radius=3 px; threshold of W=0.3), and (C) threshold of W (SHARP radius=3 px; $\lambda=10^{-1.5}$).

With Partial Volume Effect (parallel to B0)

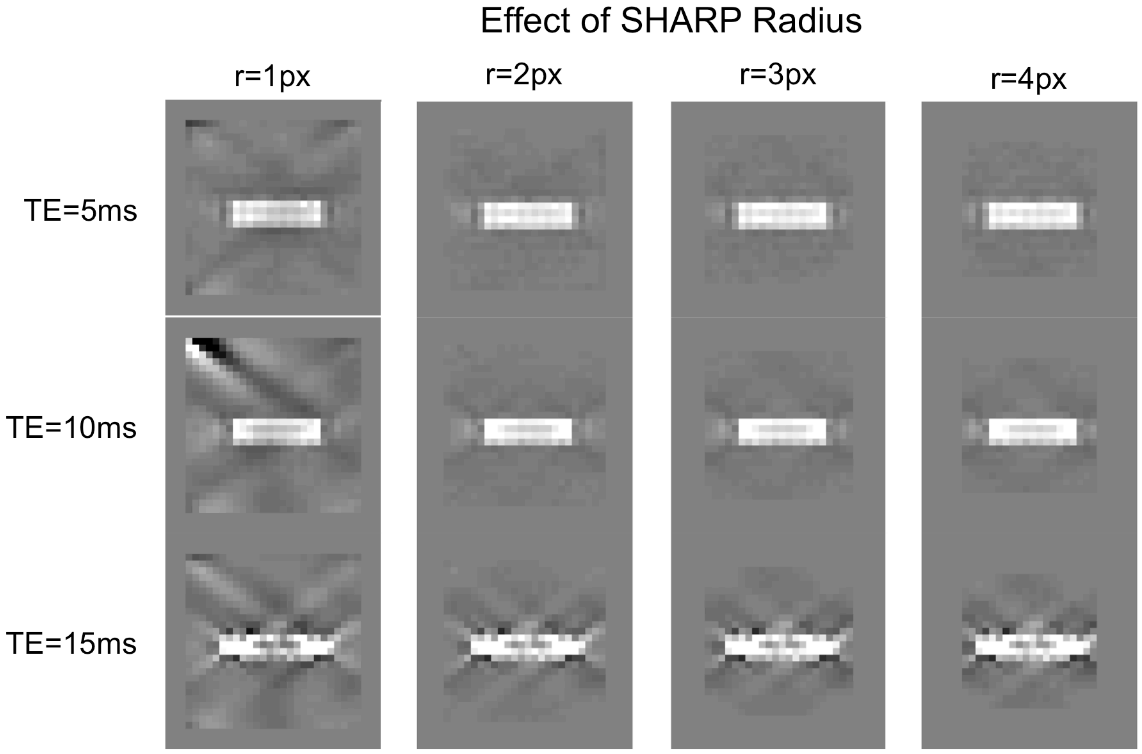

Figure P. QSMs using various SHARP radius at three different TEs. ($\lambda=10^{-1.5}$; threshold of W=0.3).

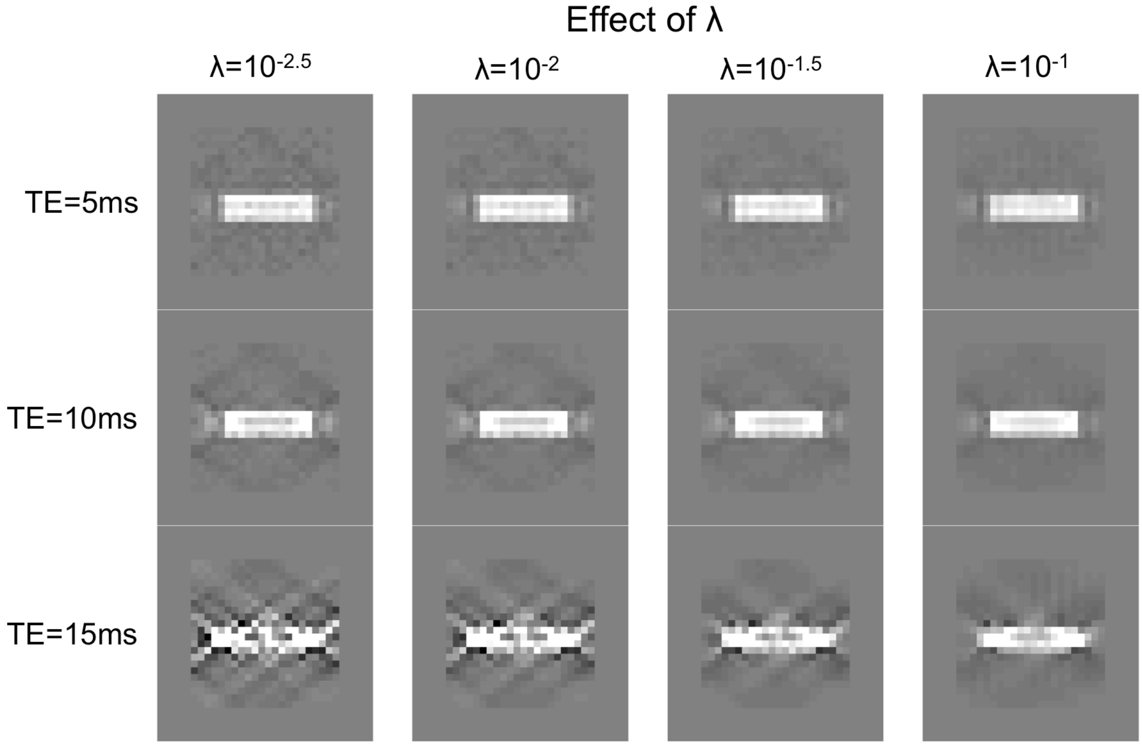

Figure Q. QSMs using various λ at three different TEs. (SHARP radius=3 px; threshold of W=0.3).

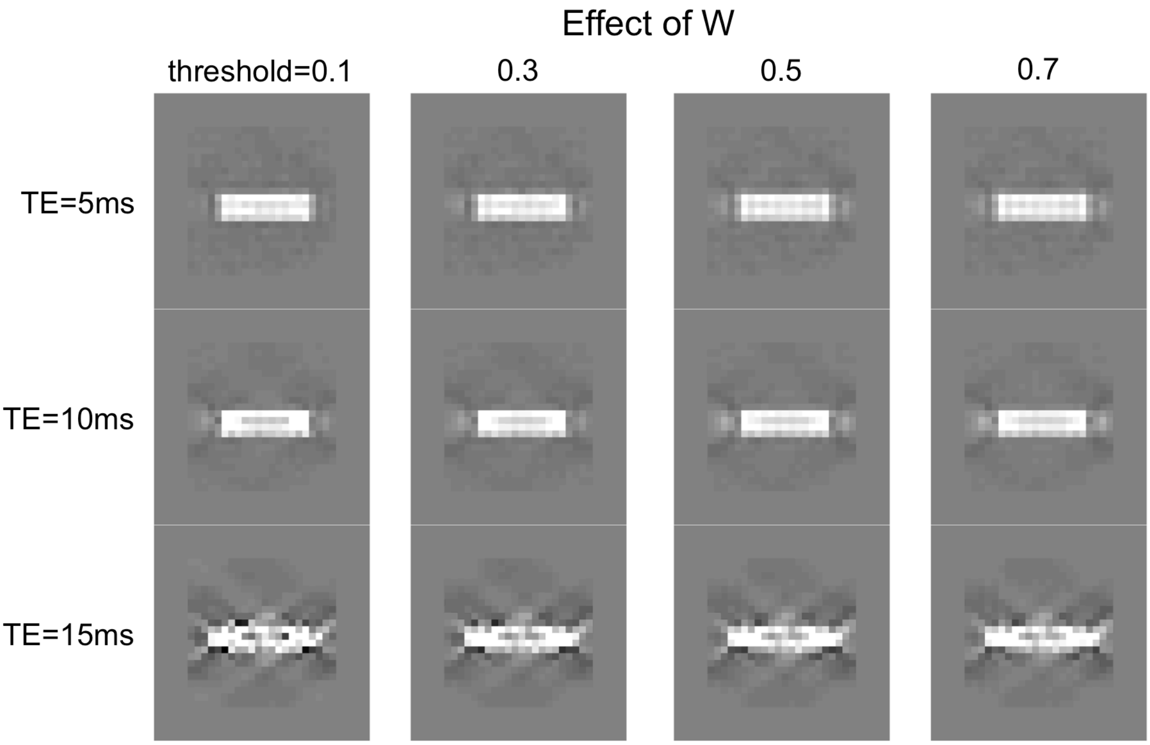

Figure R. QSMs using various thresholds of W at three different TEs. (SHARP radius=3 px; λ=$10^{-1.5}$).

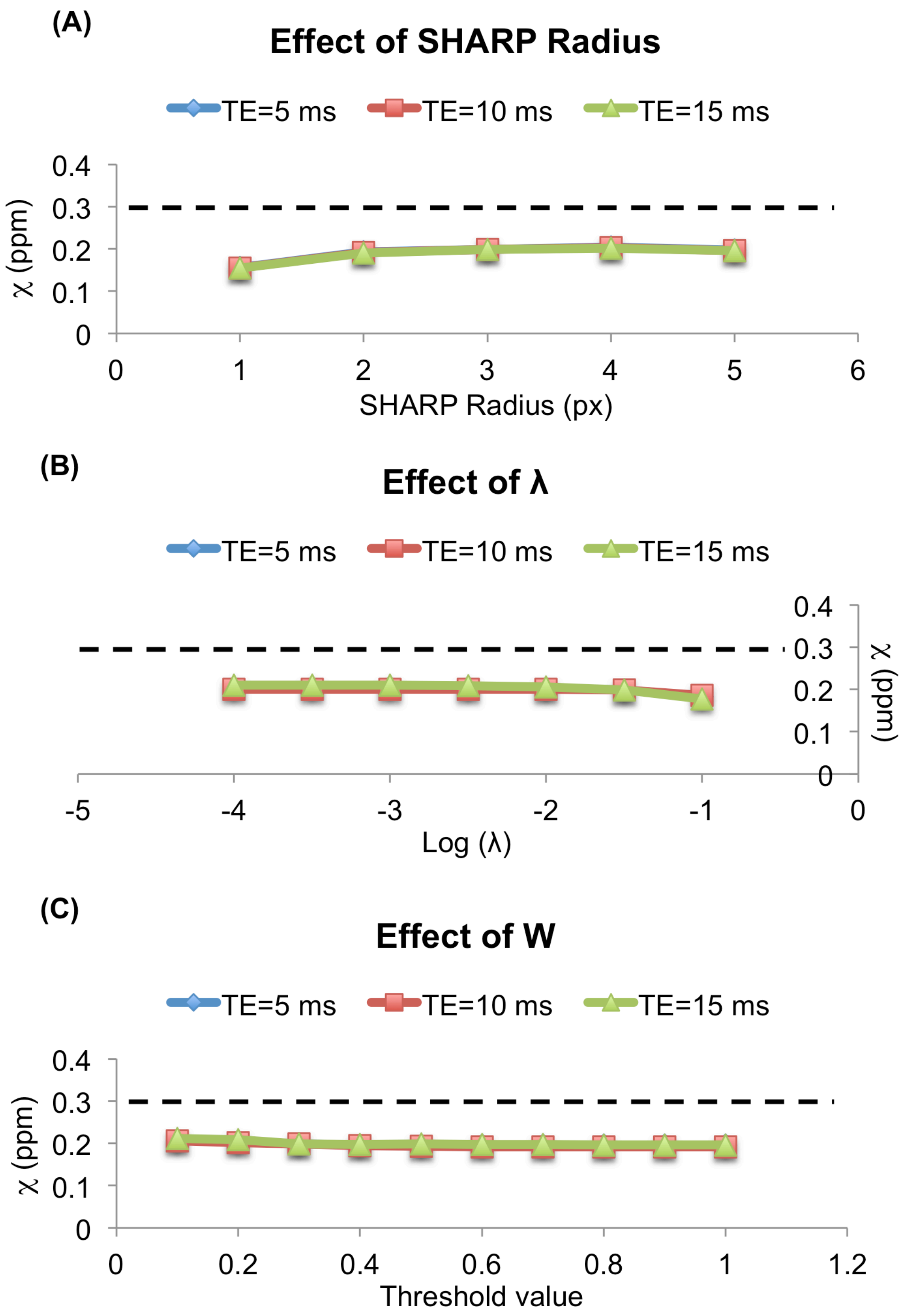

Figure S. Quantified results using various (A) SHARP radius (λ=$10^{-1.5}$; threshold of W=0.3), (B) λ (SHARP radius=3 px; threshold of W=0.3), and (C) threshold of W (SHARP radius=3 px; λ=$10^{-1.5}$).

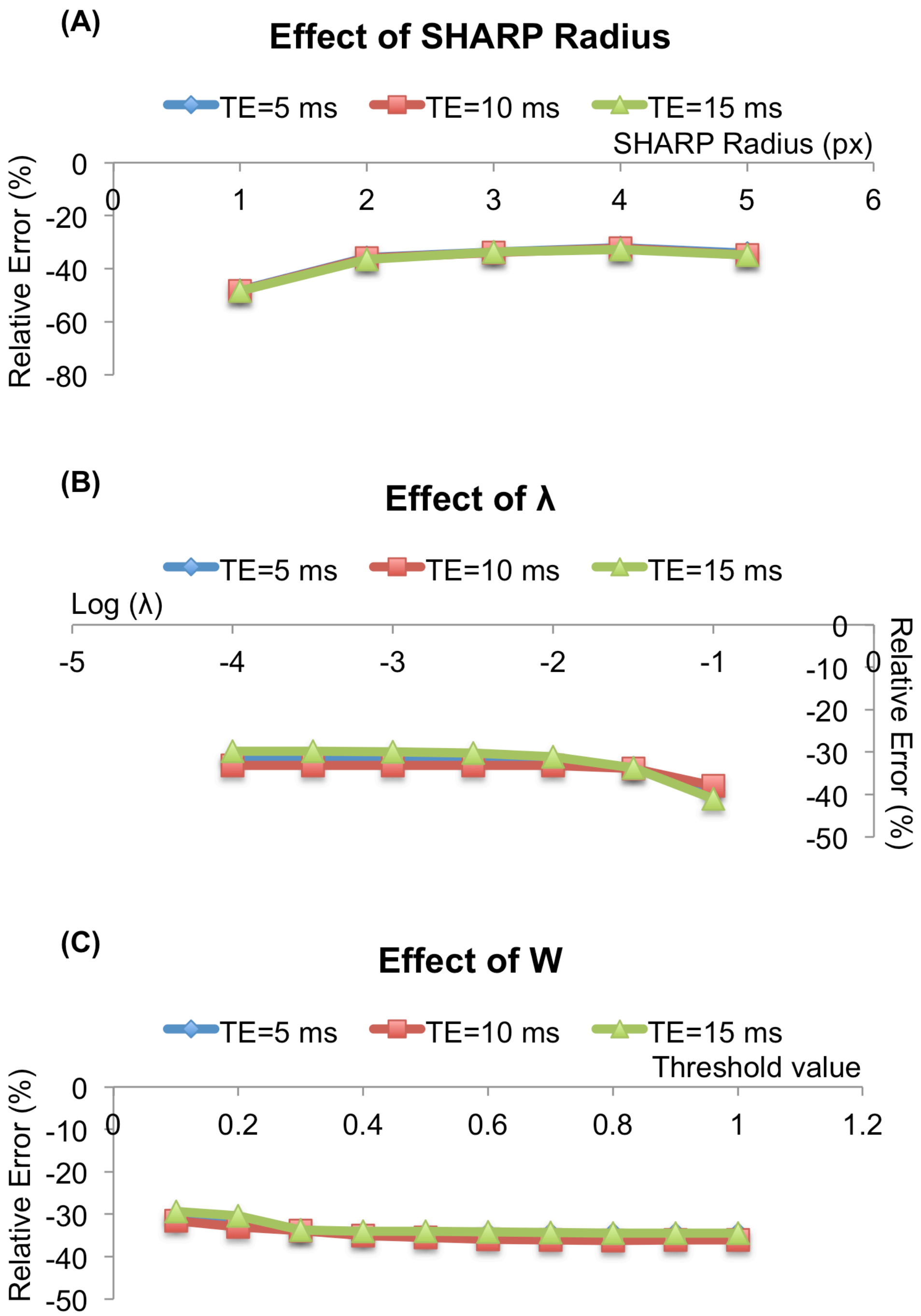

Figure T. Relative error using various (A) SHARP radius ($\lambda=10^{-1.5}$; threshold of W=0.3), (B) λ (SHARP radius=3 px; threshold of W=0.3), and (C) threshold of W (SHARP radius=3 px; $\lambda=10^{-1.5}$).

Table A. Quantified results from simulation images at three different echo times and two orientations.

	No Partial Volume Effect						With Partial Volume Effect					
Orientation to B_0	Perpendicular			Parallel			Perpendicular			Parallel		
TE (ms)	5	10	15	5	10	15	5	10	15	5	10	15
SHARP radius (px)	3	3	3	3	3	3	3	3	3	3	3	3
λ	10^{-1}	10^{-1}	10^{-1}	10^{-1}	10^{-1}	10^{-1}	$10^{-1.5}$	$10^{-1.5}$	$10^{-1.5}$	$10^{-1.5}$	$10^{-1.5}$	$10^{-1.5}$
Threshold of W	0.1	0.2	0.3	0.1	0.2	0.3	0.1	0.2	0.3	0.1	0.2	0.3
$\Delta\chi$ (ppm)	0.27	0.28	0.27	0.28	0.28	0.28	0.21	0.24	0.24	0.21	0.20	0.20
Relative Error (%)	-8.6	-7.2	-8.8	-5.5	-5.9	-5.9	-29.2	-23	-18.6	-30.9	-33	-33.8
